# Adventitial delivery of nanoparticles encapsulated with 1α, 25-dihydroxyvitamin D_3_ attenuates restenosis in a murine angioplasty model

**DOI:** 10.1038/s41598-021-84444-x

**Published:** 2021-02-26

**Authors:** Chuanqi Cai, Sreenivasulu Kilari, Chenglei Zhao, Avishek K. Singh, Michael L. Simeon, Avanish Misra, Yiqing Li, Edwin Takahashi, Rajiv Kumar, Sanjay Misra

**Affiliations:** 1 Department of Vascular Surgery, Union Hospital, Tongji Medical College, Huazhong University of Science and Technology, Wuhan, 430022 China; 2 Vascular and Interventional Radiology Translational Laboratory, Department of Radiology, Mayo Clinic, 200 First St SW, Rochester, MN 55905 USA; 3 Department of Vascular Surgery, The Second Xiangya Hospital, Central South University, Changsha, Hunan China; 4 Department of Biochemistry and Molecular Biology, Mayo Clinic, Rochester, MN USA; 5 Division of Nephrology and Hypertension, Department of Internal Medicine, Mayo Clinic, Rochester, MN USA; 6 Department of Radiology, Vascular and Interventional Radiology, Mayo Clinic, Rochester, MN USA

**Keywords:** Haemodialysis, Chronic inflammation, Nephrology

## Abstract

Percutaneous transluminal angioplasty (PTA) of stenotic arteriovenous fistulas (AVFs) is performed to maintain optimal function and patency. The one-year patency rate is 60% because of venous neointimal hyperplasia (VNH) and venous stenosis (VS) formation. Immediate early response gene X-1 (*Iex-1*) also known as *Ier3* increases in response to wall shear stress (WSS), and can cause VNH/VS formation in murine AVF. In human stenotic samples from AVFs, we demonstrated increased gene expression of *Ier3*. We hypothesized that 1α, 25-dihydroxyvitamin D_3_, an inhibitor of IER3 delivered as 1α, 25-dihydroxyvitamin D_3_ encapsulated in poly lactic-*co*-glycolic acid (PLGA) nanoparticles loaded in Pluronic F127 hydrogel (1,25 NP) to the adventitia of the stenotic outflow vein after PTA would decrease VNH/VS formation by reducing *Ier3* and chemokine (C–C motif) ligand 2 (*Ccl2*) expression. In our murine model of AVF stenosis treated with PTA, increased expression of *Ier3* and *Ccl2* was observed. Using this model, PTA was performed and 10-μL of 1,25 NP or control vehicle (PLGA in hydrogel) was administered by adventitial delivery. Animals were sacrificed at day 3 for unbiased whole genome transcriptomic analysis and at day 21 for immunohistochemical analysis. Doppler US was performed weekly after AVF creation. At day 3, significantly lower gene expression of *Ier3* and *Ccl2* was noted in 1,25 NP treated vessels. Twenty-one days after PTA, 1,25 NP treated vessels had increased lumen vessel area, with decreased neointima area/media area ratio and cell density compared to vehicle controls. There was a significant increase in apoptosis, with a reduction in CD68, F4/80, CD45, pro-inflammatory macrophages, fibroblasts, Picrosirius red, Masson’s trichrome, collagen IV, and proliferation accompanied with higher wall shear stress (WSS) and average peak velocity. IER3 staining was localized to CD68 and FSP-1 (+) cells. After 1,25 NP delivery, there was a decrease in the proliferation of α-SMA (+) and CD68 (+) cells with increase in the apoptosis of FSP-1 (+) and CD68 (+) cells compared to vehicle controls. RNA sequencing revealed a decrease in inflammatory and apoptosis pathways following 1,25 NP delivery. These data suggest that adventitial delivery of 1,25 NP reduces VNH and venous stenosis formation after PTA.

## Introduction

Arteriovenous fistulas (AVF) are the preferred mode of hemodialysis vascular access in end stage renal disease (ESRD) patients receiving hemodialysis^1^. In 2016, there were more than 726,331 patients with ESRD in the United States, and the incident number of ESRD patients is expected to increase by 20,000 per year^1^. Several studies have shown that the primary patency rate of AVF is poor—only 60% at one year^1^. Percutaneous transluminal angioplasty (PTA) is the first line treatment for malfunctioning AVF caused by vascular stenosis^2^. After PTA, AVF restenosis develops due to restenosis caused by venous neointimal hyperplasia (VNH) resulting in poor patency^3,4^. As a consequence, therapeutic measures are needed to improve AVF patency following PTA. There is an urgent need to develop other therapies that can be delivered locally to the vessel wall to reduce venous stenosis (VS) formation after PTA to treat stenotic hemodialysis AVFs.

Venous neointimal hyperplasia occurs because of multiple factor including inflammation, uremia, hypoxia, and shear stress leading to an increase in deposition of cellular and extracellular matrix in the intimal layer^5^. Immediate early response 3 (*Ier3*) gene expression has been shown to be involved in vascular injury by causing proliferation mediated by NF-kb pathway in response to shear stress^6^. We have previously reported that decreasing *Ier3* (Immediate Early Response 3) gene expression is linked to reduction in VNH formation in *Ier3*^−/−^ mice with AVF and CKD^7^. 1α,25(OH)_2_D_3_ reduces *Ier3* expression^8^ and inhibition of *Ier3* expression using* a* long-acting inhibitor, 1α,25(OH)_2_D_3_ poly(lactic-co-glycolic acid) (PLGA) embedded in a thermosensitive Pluronic F127 hydrogel (1,25 NP) decreases VNH/VS formation in a murine animal model^7^.

The purpose of this study was to determine whether 1,25 NP delivered to the adventitia of the outflow vein after PTA reduces subsequent development of VNH/VS formation. In the present study, we first determined the gene expression of *Ier3* in the endothelium of VS in patients with AVF and compared it to matched non-stenotic endothelial vein samples from the same patients using endovascular techniques^9^. It is known that the normal endothelium expresses *Ier3*^10^, but the expression in stenotic endothelial cells from human AVFs is unknown. The gene and protein expression of IER3 was determined in outflow veins removed from mouse model of PTA^4^. We decreased *Ier3* gene expression using 1,25 NP and the changes in VNH/VS were determined using histomorphometric analysis, immunohistologic changes, with hemodynamic changes assessed with Doppler ultrasound, and gene expression^4^. Finally, we performed unbiased whole transcriptomic RNA sequencing with differential gene expression analysis on outflow veins after PTA treated with 1,25 NP compared to controls^11^. This paper provides the rationale for performing large animal studies using adventitial delivery of 1,25 NPs in preventing VNH after PTA for the treatment of stenotic hemodialysis AVFs.

## Results

### *Ier3* expression was significantly Increased in stenotic clinical AVFs by endovascular biopsy and murine PTA treated vessels

**Figure 1:**
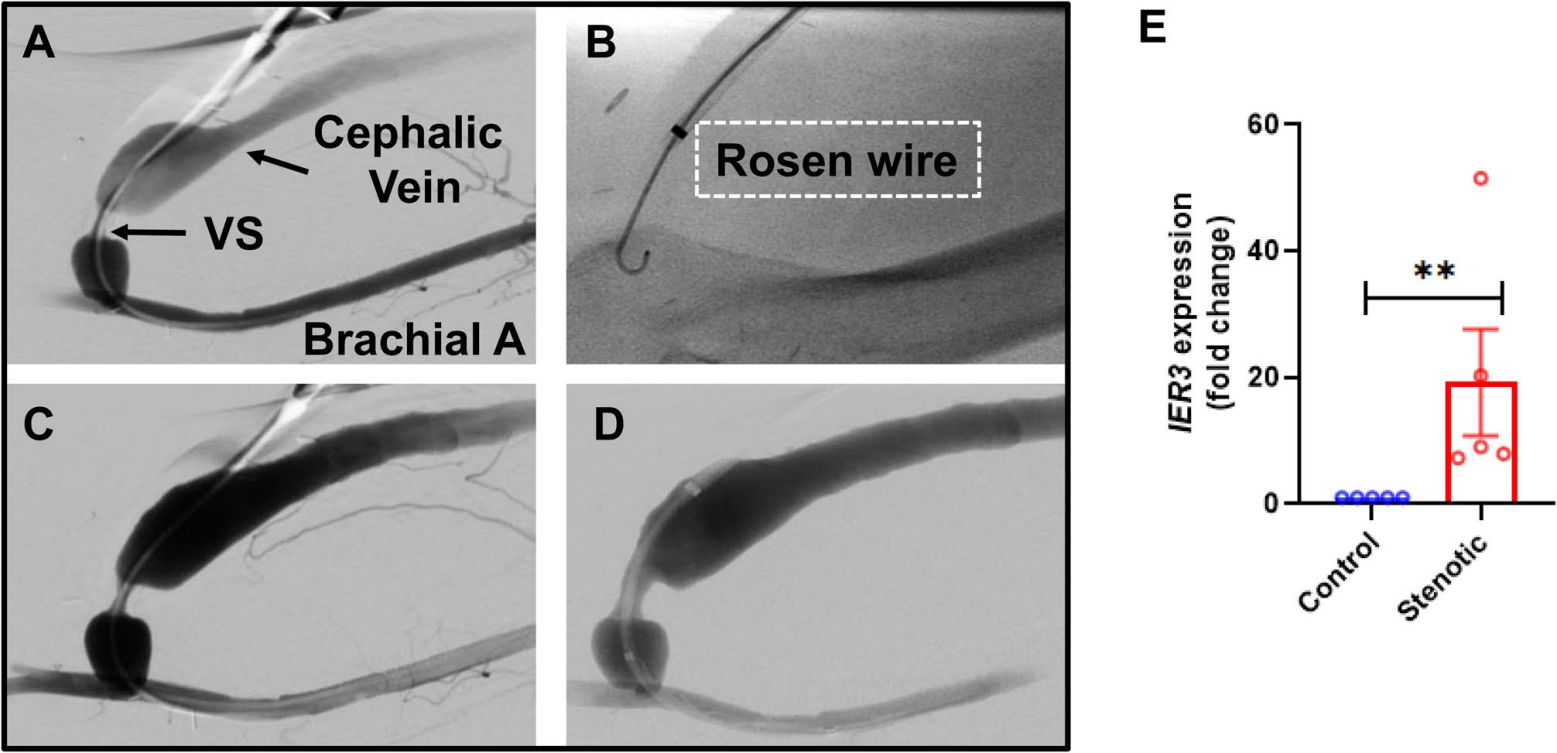
Increased *Ier3* gene expression in stenotic hemodialysis vascular access. (**A**) Representative angiogram showing stenosis at the anastomosis of the brachial artery to cephalic vein. (**B**) The insertion of Rosen wire across stenosis to sample the stenosis. (**C**) Angiogram of the stenosis after venous sampling. (**D**) Angiogram of the stenosis after angioplasty. (**E**) There is a significant increase in the average gene expression of *Ier3* of stenotic AVFs compared to controls. Each data point in the scatter plot bar graph represents the mean ± SEM of 5 patients. Mann Whitney test was performed. Significant differences between groups are indicated *p < 0.05.

The gene expression of *Ier3* in the endothelium of stenotic AVFs is unknown. We performed endovascular biopsy of human stenotic AVFs prior to PTA procedures (N = 5, 4 males, 1 female, average age = 66 ± 7 years old, Supplementary Table 1)^12^. Samples were obtained from non-stenotic veins of AVFs of each patient to serve as their own control. There was a significant increase in the mean gene expression of *Ier3* in endothelial cells removed from stenotic AVFs compared to controls (stenotic AVFs: 19.2 ± 18.8, control: 1.00 ± 0.04, average increase: 1920%, *p* < 0.01, Fig. 1E).

The gene and protein expression of IER3 is unknown after PTA. This was assessed in the murine PTA model in which an AVF stenosis is treated with PTA in mice with CKD (Supplementary Fig. 1). We determined the gene and protein expression of IER3 in PTA treated graft veins (GV) compared to contralateral jugular veins (CV) at 3 and 21 days (Supplementary Fig. 2). At day 3, there was a significant increase in the average gene expression of *Ier3* (GV: 1.61 ± 0.09; CV: 0.95 ± 0.10; average increase: 69%, *p* < 0.01, Supplementary Fig. 2E) and *Ccl2* (GV: 2.09 ± 0.37; CV: 0.80 ± 0.14; average increase: 161%, *p* < 0.01, Supplementary Fig. 2F). At day 21, there was a significant increase in IER3 (Supplementary Fig. 2A), CCL2 (Supplementary Fig. 2B) and CD68 (Supplementary Fig. 2C) staining with a decrease α-SMA (Supplementary Fig. 2D) staining in GVs compared to CVs. Semiquantitative analysis demonstrated that there was a significant increase in the average IER3 (GV: 5.95 ± 1.05; CV: 0.003 ± 0.003; average increase: 198,233%, *p* < 0.001, Supplementary Fig. 2G), CCL2 (GV: 1.66 ± 0.24; CV: 0.19 ± 0.03; average increase: 774%, *p* < 0.001, Supplementary Fig. 2H) and CD68 (GV: 9.29 ± 1.70; CV: 1.30 ± 0.07; average increase: 615%, *p* < 0.001, Supplementary Fig. 2I) staining with a decrease in the α-SMA (GV: 2.85 ± 0.50; CV: 4.85 ± 0.36; average decrease: 41%, *p* < 0.01, Supplementary Fig. 2J) in PTA treated vessels compared to CVs.

**Figure 2:**
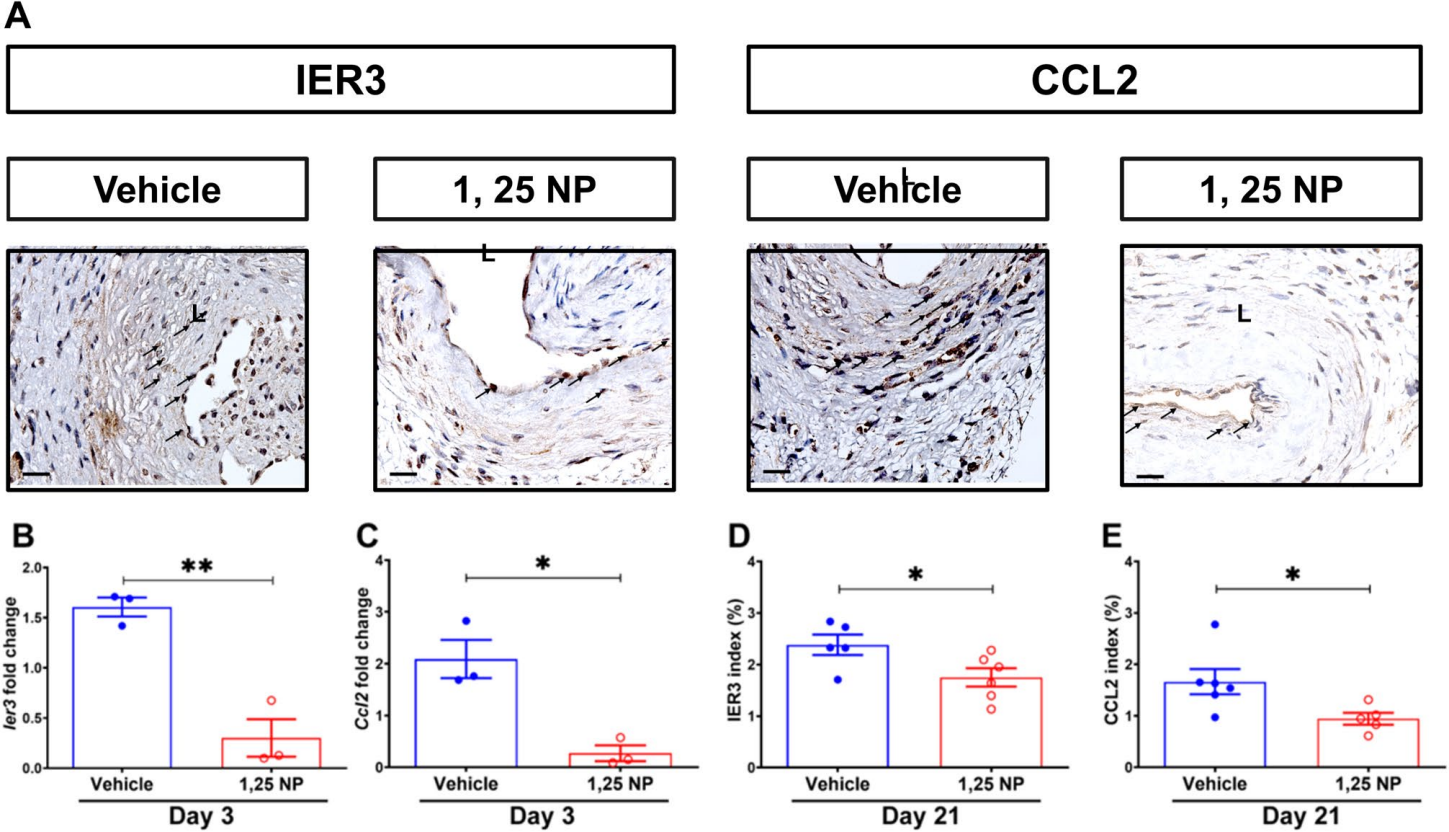
Decreased IER3 and CCL2 gene and protein expression in PTA plus 1,25 NP treated vessels. (**A**) Representative IER3 and CCL2 staining at day 21 in the 1,25 NP treated outflow graft veins and vehicle control veins, respectively. (**B**, **C**) At day 3, there was a significant decrease in the average gene expression of *Ier3* and *Ccl2* of 1,25 NP treated GVs compared to vehicle controls, respectively. (**D**, **E**) Semiquantitative analysis demonstrated significant decreases in the average IER3 and CCL2 indexes in 1,25 NP treated vessels compared to controls. Each data point in the scatter plot bar graph represents the mean ± SEM of 3–6 animals. Unpaired *t-*test was performed. Significant differences between groups are indicated *p < 0.05, **p < 0.01, ***p < 0.001. Cells staining positive for IER3 and CCL2 are brown staining. *L* lumen, *ADV* adventitial; solid arrows, positive cells. Scale bar is 50 μm.

### Serum changes in kidney and liver function in 1,25 NP and vehicle control animals

We assessed the changes in blood urea nitrogen (BUN), creatinine, ALT, AST, total bilirubin, and calcium after local delivery of 1,25 NP or vehicle control to the outflow veins by measuring. There was no significant difference in these assessments at day 3 and 21 after 1,25 NP delivery compared to vehicle controls (Supplementary Table 2).

### *Ier3* gene and protein expression is significantly decreased in 1,25 NP treated vessels compared to vehicle controls

In order to examine the therapeutic effect of IER3 inhibition in PTA treated vessels, we delivered 1,25 NP or vehicle to the adventitial layer of the outflow vein immediately after PTA (Supplementary Fig. 1 and Fig. 2). At day 3, the average gene expression of *Ier3* and *Ccl2* was significantly decreased in the 1,25 NP treated vessels compared to vehicle controls (*Ier3*: 1,25 NP: 0.30 ± 0.15, vehicle: 1.61 ± 0.08, average decrease: 81%, *p* < 0.01, Fig. 2B; *Ccl2*: 1,25 NP: 0.27 ± 0.12, vehicle: 2.09 ± 0.30, average decrease: 87%, *p* < 0.05, Fig. 2C). At day 21, there was a significant decrease in the average IER3 (1,25 NP: 1.75 ± 0.18, vehicle: 2.38 ± 0.20, average decrease: 26%, *p* < 0.05, Fig. 2A, D) and CCL2 (1,25 NP: 0.94 ± 0.11, vehicle: 1.66 ± 0.24, average decrease: 43%, *p* < 0.05, Fig. 2A, E) staining in 1,25 NP treated vessels compared to vehicle controls. We next performed co-staining of IER3 with CD68, FSP-1, and α-SMA on tissue sections from day 21 vessels for both groups (Supplementary Figs. 3A–F). We observed that IER3 staining was decreased in the CD68 (+) and FSP-1 (+) cells in the 1,25 NP treated vessels compared to vehicle controls.

**Figure 3:**
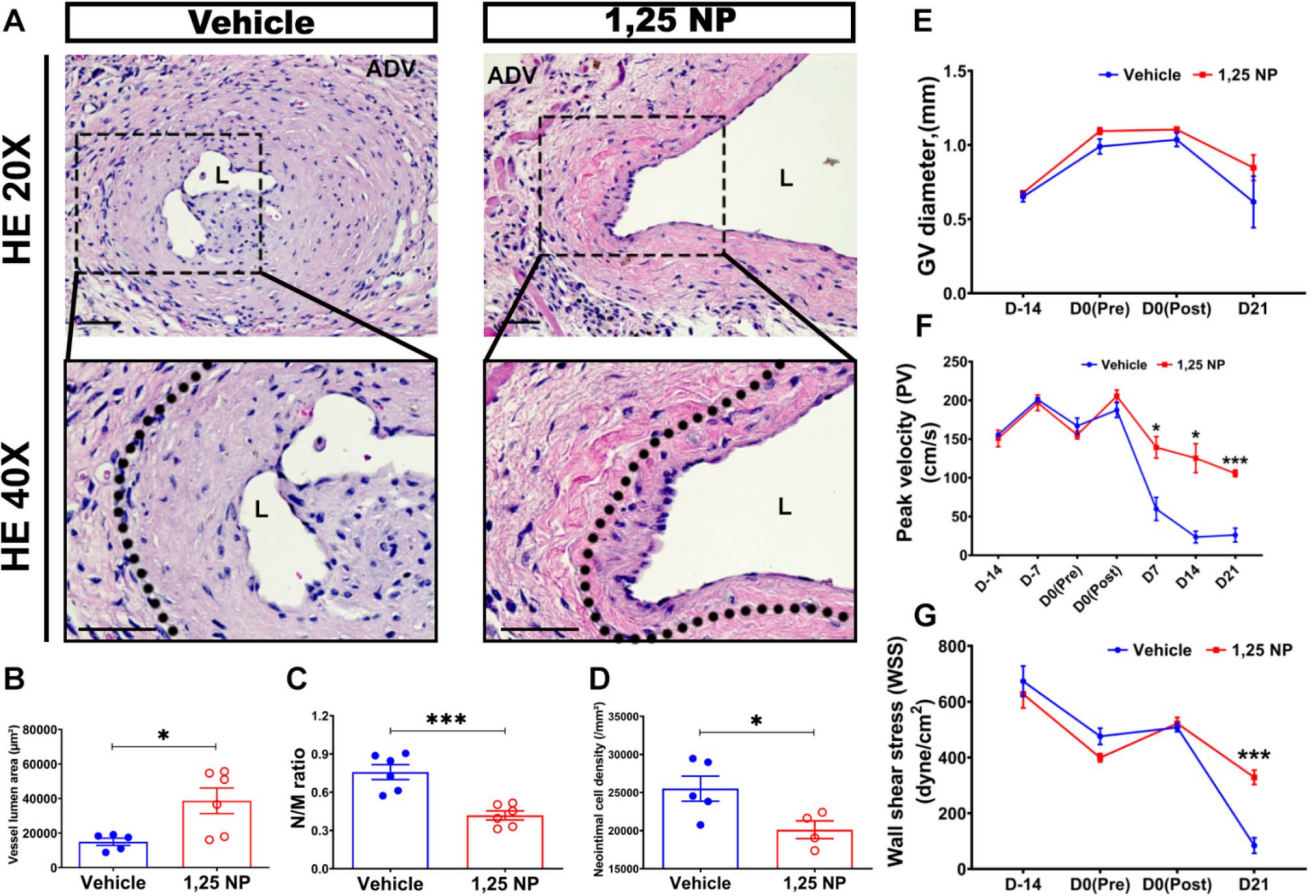
Murine outcomes in 1,25 NP treated vessels. (**A**) Representative H and E sections from vehicle and 1,25 NP treated AVFs at day 21, respectively. In 1,25 NP AVFs compared to vehicle controls, there was significant increase in the average lumen vessel area (**B**), but decrease in N/MA ratio (**C**) and neointimal cell density (**D**). There was increase in the average outflow venous diameter in 1,25 NP group mice (**E**). At day 21 after PTA plus 1,25 NP delivery, there was a significant increase in the average peak velocity (**F**) and wall shear stress (**G**) compared to controls. Each time point represents the mean ± SEM of 5–6 animals. Two-way ANOVA with Bonferroni’s correction was performed in (**E**–**G**). (**B**–**D**) were performed with unpaired *t* test. Significant differences between vehicle and 1,25 NP treated vessels are indicated *p < 0.05, **p < 0.01. *N/M* neointima area/media area; *ADV* adventitia, *L* lumen. Scale bar is 50 μm.

### Positive vascular remodeling of 1,25 NP treated vessels compared to vehicle controls

The outflow venous patency and peak velocity (PV) were determined weekly by using Doppler ultrasound. There was improved patency observed in 1,25 NP treated vessels (89%) compared to vehicle control group (78%). Twenty-one days after PTA, histomorphometric analysis was performed on paraffin embedded H and E stained sections (Fig. 3A). The average lumen vessel area of 1,25 NP treated vessels was significantly increased compared to vehicle controls (1,25 NP: 38,697.37 ± 7401.21 μm^2^, vehicle: 14,967.31 ± 2099.02 μm^2^, average increase: 259%, *p* < 0.05, Fig. 3B). The average neointima/media area ratio (N/M) was significantly decreased in the 1,25 NP treated vessels compared to vehicle controls (1,25 NP: 0.42 ± 0.04, vehicle: 0.76 ± 0.06, average decrease: 45%, *p* < 0.001, Fig. 3C) with no difference in the neointima area. Additionally, the average cell density in the neointima was significantly reduced in the 1,25 NP treated vessels compared to vehicle controls (1,25 NP: 20,115.90 ± 1161.25 /mm^2^, vehicle: 25,511.37 ± 1645.19 /mm^2^, average decrease: 21%, *p* < 0.05, Fig. 3D). The diameter of outflow veins was measured intraoperatively after AVF placement, at pre- and post-PTA, and at sacrifice (Fig. 3E). Twenty-one days after PTA, there was no difference in the average diameter of the outflow vein between the 1,25 NP group and vehicle control group (Fig. 3E).

### There is an increase in the average peak velocity (PV) and wall shear stress (WSS) of 1,25 NP treated vessels compared to vehicle controls

Peak velocity of the blood through the outflow vein was determined using Doppler US. Before PTA, there was no significant difference in the average PV between the two groups (Fig. 3F). At day 21, after PTA, the average PV was significantly higher in the 1,25 NP treated vessels compared to vehicle controls (1,25 NP: 105.73 ± 4.38 cm/s, vehicle: 26.07 ± 8.91 cm/s, average increase: 406%, *p* < 0.001, Fig. 3F). At day 21, the average WSS was significantly higher in the 1,25 NP treated vessels compared to vehicle controls (1,25 NP: 326.58 ± 20.86 dynes/cm^2^, vehicle: 83.84 ± 27.63 dynes/cm^2^, average increase: 390%, *p* < 0.001, Fig. 3G).

### There was an increase in the contractile SMCs with a decrease in the synthetic phenotype with a decrease in FSP-1 in 1,25 NP treated vessels compared to vehicle controls

**Figure 4:**
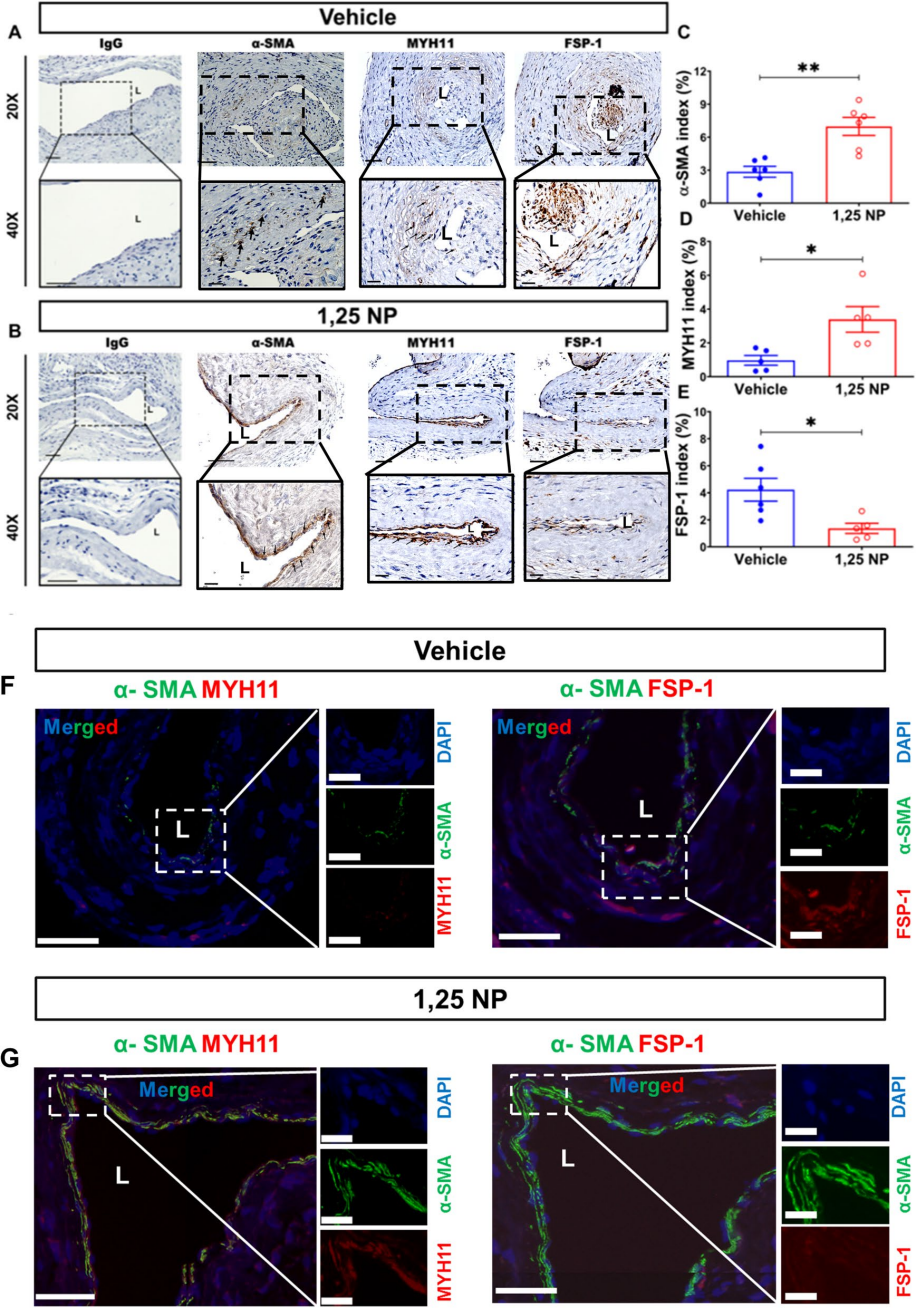
α-SMA, MYH11, and FSP-1 staining in 1,25 NP treated vessels. (**A** and **B**) Representative images for negative control (IgG), α-SMA, MYH11, and FSP-1 staining are shown in vehicle controls and 1,25 NP treated vessels. (**C**–**E**) Semiquantitative analysis showed a significant increase in the average α-SMA (*p* < 0.05), MYH11 (*p* < 0.05), but decrease in FSP-1 (*p* < 0.05) indices in 1,25 NP treated vessels compared to vehicle controls, respectively. (**F**, **G**) Co-staining of α-SMA (+)/ MYH11 (+) and FSP-1 (+)/MHY11 (+) staining was performed in both groups at day 21. There are more α-SMA (+)/MYH11 (+) cells in 1,25 NP vessels compared to vehicle controls. Each data point in the scatter plot bar graph represents the mean ± SEM of 5–6 AVF mice. Unpaired *t-*test was performed. Significant differences are indicated *p < 0.05. (**A**, **B**) Cells staining positive for α-SMA, MYH11 and FSP-1 are brown staining. (**F**, **G**) Green color indicates positive α-SMA staining. Red color indicates MYH11 or FSP-1 positive staining. Blue color indicates positive staining for nuclei. *ADV* adventitia, *L* lumen; solid arrows, positive cells; dashed red line, positive stained area. Scale bar is 50 μm.

Venous neointimal hyperplasia is characterized histologically by the accumulation of smooth muscle cells (SMCs) in the intima and media^5^. The presence of SMCs was determined using α-SMA. There were increased cells staining positive for α-SMA in the neointima of the outflow vein of the 1,25 NP treated vessels compared to vehicle group (Fig. 4A) with a significant increase in the average α-SMA index (1,25 NP: 6.98 ± 0.82, vehicle: 2.85 ± 0.50, average increase: 245%, *p* < 0.01, Fig. 4C). We wanted to assess if the α-SMA increase was due to contractile or synthetic MSCs. MHY11 staining was performed and this showed that there was decrease in the MYH11 index in 1,25 NP vessels compared to vehicle controls (1,25 NP: 3.39 ± 0.76, vehicle: 0.97 ± 0.29, average increase: 349%, *p* < 0.05, Fig. 4D). In order to determine the presence of contractile vascular SMCs, we performed double staining for α-SMA (+)/MHY11 (+). There was an increase in the co-staining for α-SMA (+)/MYH11 (+) cells in 1,25 NP treated vessels (Fig. 4G, left panel) compared to vehicle controls (Fig. 4F, left panel). These data suggest that there is more contractile VSMC in 1,25 NP treated vessels compared to vehicle treated vessels.

The presence of synthetic SMC phenotype was determined using vimentin, MMP-2, and MMP-9 staining (Supplementary Fig. 5). The synthetic phenotype is associated with increased fibrosis due to collagen secretion. In 1,25 NP treated vessels compared to control vehicles, there was a significant reduction in vimentin (1,25 NP: 17.5 ± 2.31, vehicle: 29 ± 2.93, average decrease: 40%, *p* < 0.05, Supplementary Fig. 5C), MMP-2 (1,25 NP: 2.81 ± 0.65, vehicle: 5.90 ± 0.44, average decrease: 52%, *p* < 0.05, Supplementary Fig. 5D), and MMP-9 staining (1,25 NP: 0.53 ± 0.12, vehicle: 1.16 ± 0.08, average decrease: 54%, *p* < 0.01, Supplementary Fig. 5E). These data suggest that there is less synthetic VSMC in 1,25 NP treated vessels compared to vehicle treated vessels.

**Figure 5:**
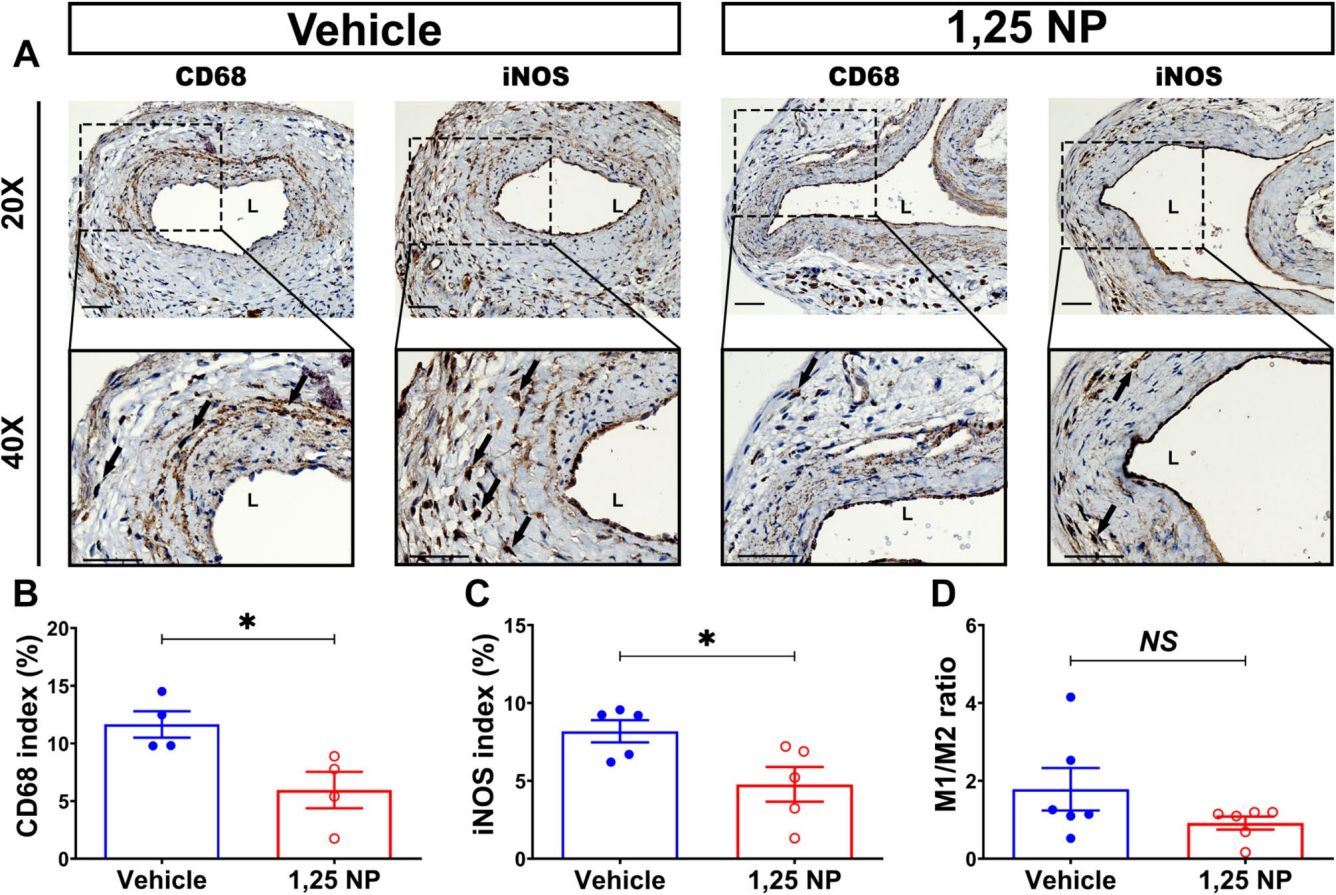
Inflammatory cells staining in 1,25 NP treated vessels. (**A**) Representative slides for negative control (IgG), CD68, and iNOS staining are shown in vehicle controls and 1,25 NP treated vessels. There is increased CD68 and iNOS (+) cells in the vessel controls compared to 1,25 NP treated vessels. (**B**–**D**) Semi-quantitative analysis showed a significant decrease in the average CD68 (*p* < 0.05) and iNOS (*p* < 0.05) indices, but no difference in the average M1/M2 (*p* > 0.05) index in 1,25 NP treated vessels compared to vehicle controls. Each data point in the scatter plot bar graph represents the mean ± SEM of 4–6 AVF mice. Unpaired *t-*test was performed. Significant differences are indicated *p < 0.05. Cells staining positive for CD68 and iNOS staining are brown. *ADV* adventitia, *L* lumen; solid arrows, positive cells. Scale bar is 50 μm.

Venous stenosis formation can occur because of differentiation of fibroblasts to SMCs^13^. We assessed the presence of fibroblasts by staining for fibroblasts specific protein-1 (FSP-1). There were more cells staining positive for FSP-1 in the vehicle control tissues compared to 1,25 NP treated vessels (Fig. 4A, B). There was a significant reduction in the average FSP-1 index of 1,25 NP treated vessels compared to vehicle controls (1,25 NP: 1.36 ± 0.38, vehicle: 4.23 ± 0.84, average decrease: 68%, *p* < 0.05, Fig. 4E). We performed staining for fibroblast activation protein-1 (FAP-1) to determine if the fibroblasts were activated and observed no difference in the staining between both groups (Supplementary Fig. 4). In order to determine if FSP-1 cells also colocalized to α-SMA, we performed double staining for α-SMA (+)/FSP-1 (+) cells. There was no difference in the co-staining for FSP-1 (+)/α-SMA (+) cells in 1,25 NP treated vessels (Fig. 4G, right panel) compared to the vehicle controls (Fig. 4F, right panel).

### Adventitial delivery of 1,25 NP decreases inflammation

RT-PCR data showed a decrease in the gene expression of *Ccl2* and this has been linked to inflammation (Fig. 2C). We performed CD68, F4/80, CD45, iNOS (M1), and Arg-1 (M2) staining to determine the changes in vascular inflammation after 1,25 NP treatment compared to vehicle controls. There were more CD68 and iNOS (+) cells in the vehicle controls compared to 1,25 NP treated vessels (Fig. 5A). The average CD68 staining (1,25 NP: 5.96 ± 1.58, vehicle: 11.65 ± 1.14, average decrease: 49%, *p* < 0.05, Fig. 5B), F4/80 staining (1,25 NP: 0.88 ± 0.1, vehicle: 2.57 ± 0.53, average decrease: 66%, *p* < 0.05, Supplementary Fig. 6C) and CD45 staining (1,25 NP: 0.72 ± 0.09, vehicle: 4.08 ± 1.21, average decrease: 82%, *p* < 0.01, Supplementary Fig. 6D) were significantly decreased in 1,25 NP treated vessels compared to control vehicles. Because F4/80, CD45, or CD68 (+) cells can differentiate into MHY11 (+) cells, we performed costaining for CD68(+)/MHY11(+), F4/80(+)/MHY11(+), or CD45(+)/MHY11(+) (Supplementary Fig. 7). We did not observe differentiation of F4/80, CD45, or CD68 into MHY11(+) cells.

**Figure 6:**
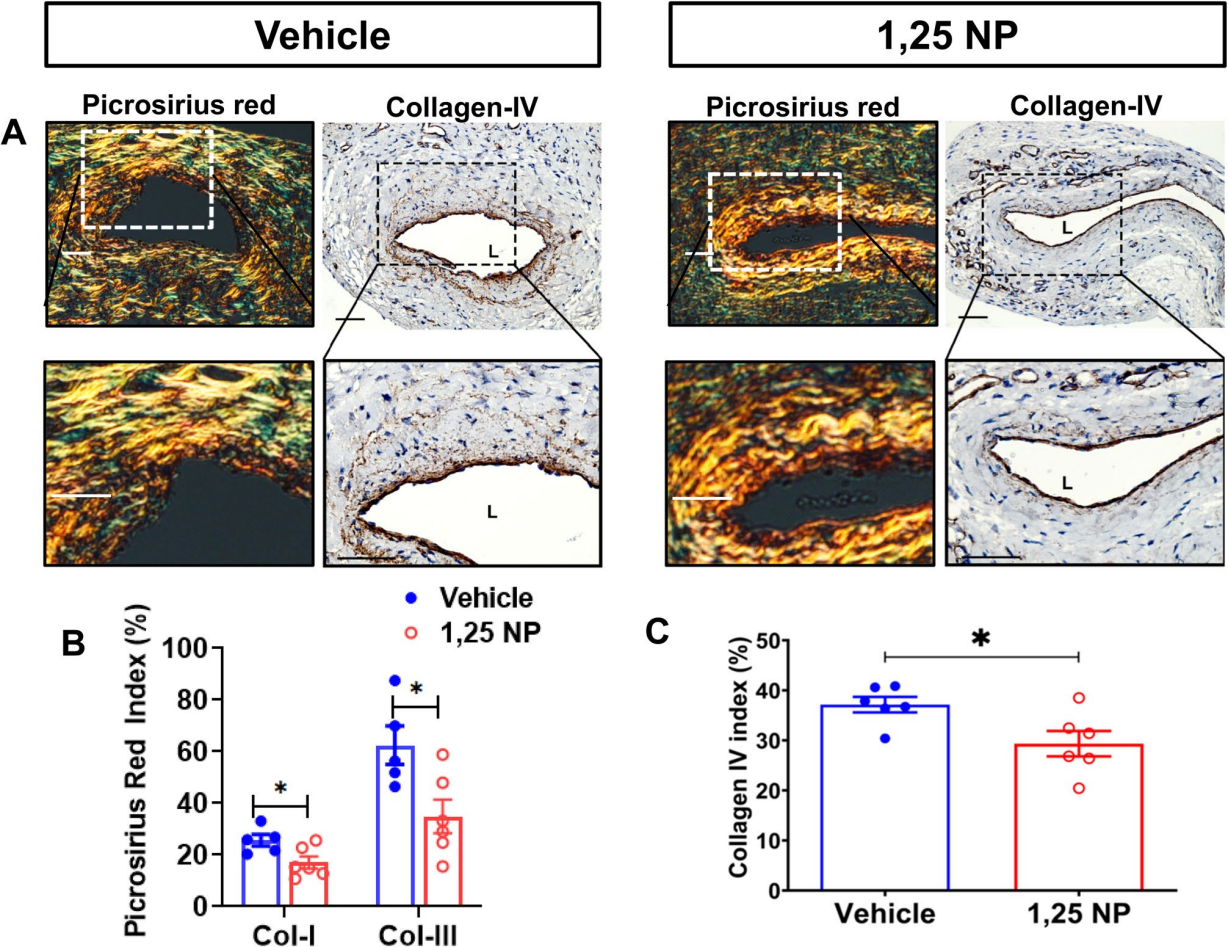
Fibrosis staining in 1,25 NP treated vessels. (**A**) Representative slides for Picrosirius red and collagen IV staining. Picrosirus red staining was visualized under polarized light, which distinguish collagen-I (yellow color) and collagen-III (green color). There is more collagen deposition in the media and neointimal area of vehicle controls compared to 1,25 NP treated vessels. (**B**) Semi-quantitative analysis showed a significant decrease in the average collagen-I and collagen-III (*p* < 0.05) indices in 1,25 NP treated vessels compared to vehicle controls. (**C**) Semi-quantitative analysis showed a significant decrease in the average collagen IV (*p* < 0.05) index in 1,25 NP treated vessels compared to vehicle controls. Each data point in the scatter plot bar graph represents the mean ± SEM of 5–6 AVFs. Unpaired *t-*test was performed. Significant differences are indicated *p < 0.05. *ADV* adventitia, *L* lumen. Scale bar is 50 μm.

**Figure 7:**
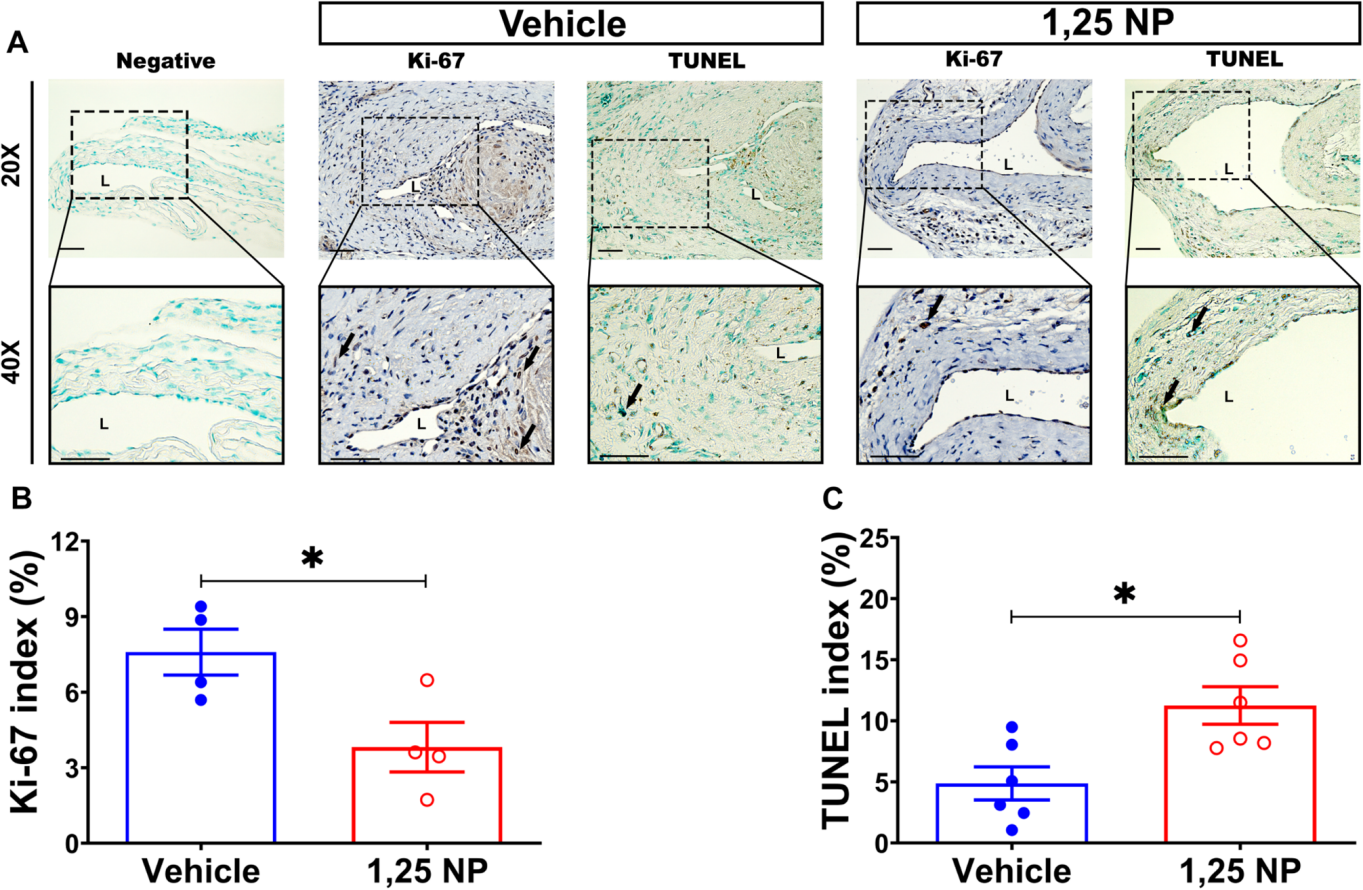
Proliferation and apoptosis staining in 1,25 NP treated vessels. (**A**) Representative slides for negative control, Ki-67 and TUNEL staining. There is more proliferation and less apoptosis in the vehicle controls compared to 1,25 NP treated vessels. (**B**, **C**) Semi-quantitative analysis demonstrated a significant decrease in the average Ki-67 index (*p* < 0.05) and increase in the average TUNEL index (*p* < 0.05) in 1,25 NP treated vessels compared to vehicle controls. Each data point in the scatter plot bar graph represents the mean ± SEM of 4–6 AVF mice. Unpaired *t-*test was performed. Significant differences are indicated *p < 0.05. (**A**) Positive staining is brown color. *L* lumen; black arrows indicate positive cells. Scale bar is 50 μm.

We determined M1 staining by performing iNOS staining and observed that the average iNOS staining (1,25 NP: 4.77 ± 1.12, vehicle: 8.18 ± 0.71, average decrease: 42%, *p* < 0.05, Fig. 5C) were significantly decreased in the 1,25 NP treated vessels compared to vehicle controls at day 21. There was no significant difference for Arg-1 staining at day 21 (Supplementary Fig. 4). We determined the average M1/M2 ratio in the 1,25 NP treated vessels compared to vehicle controls and there was no difference (Fig. 5D).

Because local delivery of 1,25 NP delivery caused a transient increase in serum calcium concentration, we assessed the presence of calcification by performing Alizarin Red staining. We observed no evidence of calcification in both groups (Supplementary Fig. 4).

### There was reduced collagen staining in 1,25 NP treated vessels

Increased fibrosis has been identified in failed AVF specimens and experimental rodent PTA model^11^. We performed Picrosirius red, collagen, and Masson’s trichrome staining to determine the fibrotic changes 21 days after PTA in 1,25 NP treated vessels and in vehicle controls. Most of the Picrosirius Red and collagen IV staining was observed in the neointima and media area in both groups (Fig. 6A). Picrosirius red staining indicated that there is a significant reduction in collagen 1 (yellow) and There was a significant reduction in the average collagen 1 staining in the 1,25 NP treated vessels compared to vehicle controls (1,25 NP: 17.03 ± 2.39, vehicle: 25.6 ± 2.26, average decrease: 33%, *p* < 0.05, Fig. 6B) and average collagen 3 staining (green) in the 1,25 NP treated vessels compared to vehicle controls (1,25 NP: 34.9 ± 6.48, vehicle: 62.5 ± 7.39, average decrease: 44%, *p* < 0.05). There was a significant reduction in the average collagen IV staining in the 1,25 NP treated vessels compared to vehicle controls (1,25 NP: 29.34 ± 2.53, vehicle: 37.13 ± 1.55, average decrease: 21%, *p* < 0.05, Fig. 6C). Finally, we performed Masson’s trichrome staining and observed a significant reduction in 1,25 NP treated vessels compared to vehicle controls (1,25 NP: 23.3 ± 2.8, vehicle: 50 ± 3.8, average decrease: 53%, *p* < 0.01, Supplementary Fig. 8).

**Figure 8:**
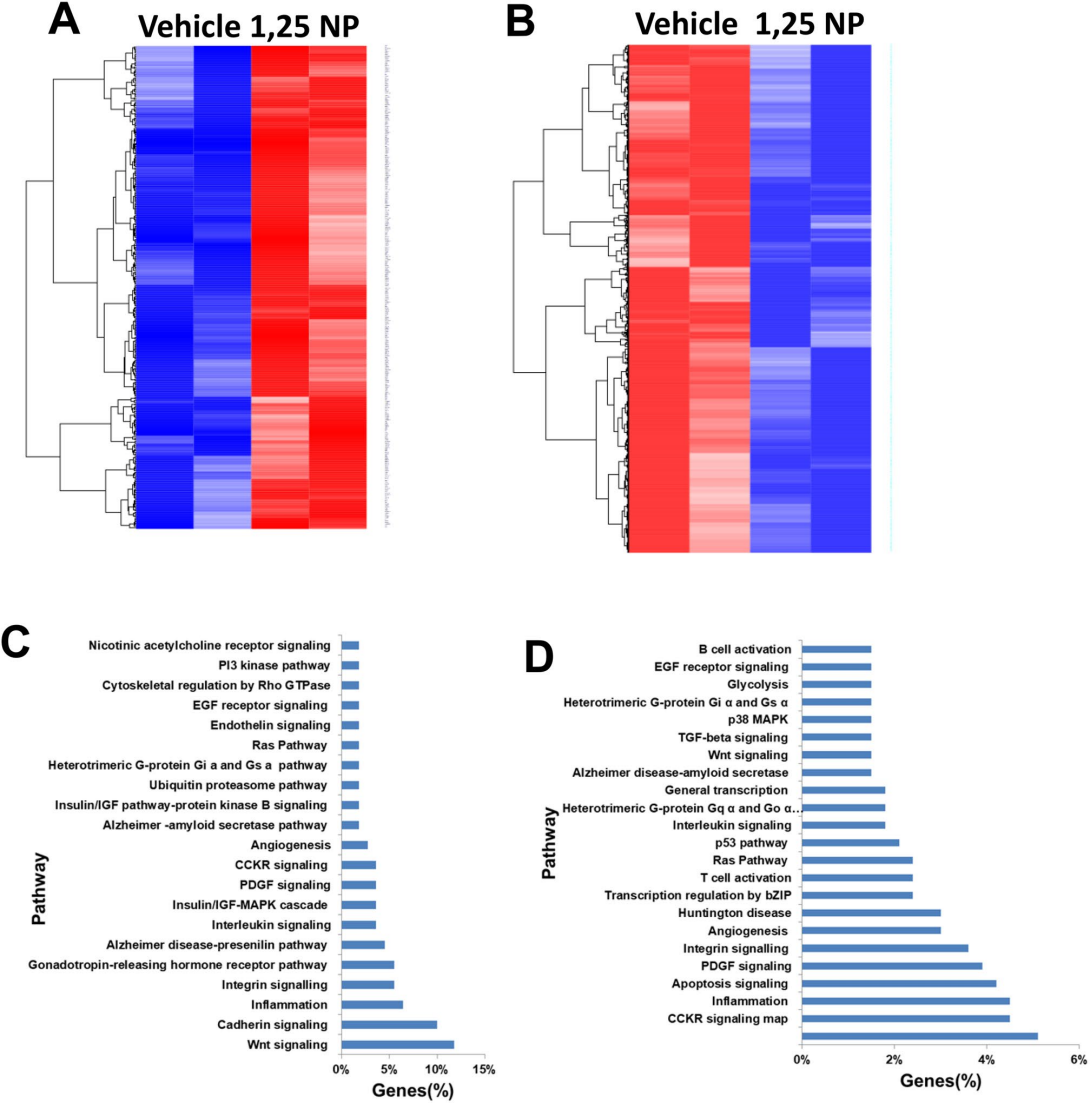
Whole transcriptome analysis using RNA sequencing with differential gene expression and Panther analyses. Unbiased whole transcriptome analysis using RNA sequencing from AVF outflow veins after PTA treated with vehicle controls compared to 1,25 NP. (**A**) Heat map depicting all the genes that are 1.5 fold up or (**B**) 0.75 fold down in outflow veins treated with 1,25 NPs compared to vehicle controls. (**C**) is Panther analysis of differentially regulated genes and in (**D**) shows analysis of downregulated genes.

### There is reduced Ki-67 with increased TUNEL staining in 1,25 NP treated vessels

Morphometric analysis showed decreased cell density after adventitial delivery of 1,25 NP. We determined whether this reduction was due to a decrease in cellular proliferation (Fig. 7A). After 1,25 NP delivery, Ki-67 (+) cells were mainly observed in the media and adventitial layers. Semiquantitative analysis for cells staining (+) for Ki-67 demonstrated a significant reduction in 1,25 NP treated vessels compared to vehicle controls (1,25 NP: 3.82 ± 0.98, vehicle: 7.59 ± 0.91, average decrease: 50%, *p* < 0.05, Fig. 7B). Next, we performed TUNEL staining in the two groups to determine the changes in apoptosis. At day 21 post PTA treatment, TUNEL (+) staining was mainly observed in the neointima and media of 1,25 NP and vehicle control vessels. The average TUNEL staining was significantly increase in the 1,25 NP treated vessels compared to vehicle controls (1,25 NP: 11.25 ± 1.54, vehicle: 4.88 ± 1.35, average increase: 331%, *p* < 0.05, Fig. 7C). We performed cleaved caspase 3 staining and semiquantification was performed. This showed a significant increase in the average activated caspase 3 staining in 1,25 NP treated vessels compared to vehicle controls (1,25 NP: 14.47 ± 0.45, vehicle: 5.71 ± 0.71, average increase: 353%, *p* < 0.05, Supplementary Fig. 9).

We wanted to identify the phenotype of the proliferative and apoptotic cells (Supplementary Figs. 10 and 11). Next, we performed co-staining of α-SMA, FSP-1 and CD68 with Ki-67 and TUNEL. There was decreased staining of Ki-67 (+)/α-SMA (+) and Ki-67 (+)/CD68 (+) cells in the 1,25 NP treated vessels (Supplementary Figs. 10D and 10F) compared to vehicle controls (Supplementary Figs. 10A and 10C). There was no difference in the co-staining of Ki-67 (+)/FSP-1 (+) cells in both groups (Supplementary Figs. 10B and 10E). With respect to TUNEL staining, there were more TUNEL (+)/FSP-1 (+) and TUNEL (+)/CD68 (+) observed in 1,25 NP treated vessels (Supplementary Figs. 11E and 11F) compared to vehicle controls (Supplementary Figs. 11B and 11C). After 1,25 NP delivery, there was a decrease in the proliferation of α-SMA (+) and CD68 (+) cells with an increase in the apoptosis of FSP-1 (+) and CD68 (+) cells compared to vehicle controls.

### Whole genome sequencing using RNA sequence with pathway analysis

We performed whole transcriptome RNA sequencing with differential gene expression analysis in 1,25 NP or vehicle treated outflow veins after PTA treatment. A heat map of the differential gene expression in 1,25 NP or vehicle controls at an average fold change > 1.5 (upregulated, Fig. 8A) or < 0.75 (downregulated, Fig. 8B) with a *P* < 0.05 is shown. There were 311 genes that were up regulated and 873 genes that were down regulated in 1,25 NP treated outflow vein compared to vehicle. Panther analysis identified that the most affected signaling pathways associated with the up regulated genes included Wnt signaling, cadherin, and inflammation (Fig. 8C). Similarly, in the down-regulated genes, Panther analysis demonstrated that the top three signaling pathways that were involved were CCKR, inflammation, and apoptosis (Fig. 8D).

## Discussion

In the present study, we demonstrated that there is increased gene expression of *IER3* in endothelial samples removed from patients with stenotic AVFs. There was increased gene and protein expression of IER3, CCL2, and CD68 staining after PTA of VS in mice with AVF and CKD compared to control veins. Adventitial delivery of 1,25 NP to the outflow veins after PTA resulted in a significant decrease in the average gene expression of *Ier3* and *Ccl2*. This was accompanied with positive vascular remodeling with as increased lumen vessel area, reduced N/M ratio and reduced cell density in the neointima at day. We observed that IER3 staining was decreased in cells staining (+) for CD68 and FSP-1 in the 1,25 NP treated vessels compared to vehicle controls. This was accompanied with a significant decrease in proliferation, inflammatory cellular phenotypes (CD68, F4/80, and CD45 (+), pro-inflammatory iNOS staining), FSP-1, fibrosis as assessed using Masson’s trichrome, collagen staining, and Picrosirius red), with an increase in contractile VSMCs with decrease in synthetic VSMCs. There was a decrease in proliferation and increase in apoptosis after 1,25 NP delivery. We assessed which cells had decreased proliferation and increased apoptosis and found that there was a decrease in the proliferation of α-SMA (+) and CD68 (+) cells with increase in the apoptosis of FSP-1 (+) and CD68 (+) cells. There was an increase in average peak velocity and WSS in the 1,25 NP treated vessels compared to vehicle controls. Finally, unbiased whole transcriptomic analysis with RNA sequencing and Panther analysis was performed to identify the mechanisms involved with 1,25 NP delivery and we observed that inflammatory and apoptotic pathways were involved.

Previous studies have shown that there is increased expression of *Ier3* and *Ccl2* in stenotic experimental and human failed AVFs, and it was localized to the adventitia^7,14^. IER3 expression has been shown to localize to the endothelium^10^. A recent study described the use of endovascular biopsy to obtain venous samples from stenotic AVFs^9^. We took advantage of this approach and determined the gene expression of *Ier3* prior to PTA treatment in patients with stenotic hemodialysis AVFs. We observed a significant increase in average gene expression of *Ier3* in stenotic endothelial cells compared to patient matched endothelial cells from non-stenotic veins from the same AVF. Because the expression of *Ier3* and *Ccl2* after PTA of AVF is unknown, we investigated the expression of *Ier3*, Ccl2 in our mouse model of PTA of VS and found that gene expression of *Ier3* and *Ccl2* increases after PTA.

Genetic deletion of *Ier3* in mice with AVF and CKD demonstrated a reduction in VS/VNH formation^7^. 1α, 25(OH)_2_D_3_ is an inhibitor of IER3^8^. Previous work from our lab has shown that *Ier3* expression is reduced with the adventitial delivery of 1,25 NP accompanied with a gene expression of *Ccl2*^7^. We postulated that adventitial delivery of 1,25 NP after PTA of outflow veins would have significant decrease in *Ier3* and *Ccl2* gene expression. We observed this and were able to localize the cells expressing IER3 by immunofluorescent technique by performing double staining for CD68 and FSP-1 cells which reside in the adventitia. There was reduced staining of IER3 in 1,25 NP treated vessels in particular cells staining positive for CD68 and FSP-1. Taken collectively, this implies that 1,25 NP treatment after PTA reduces the expression of IER3 in CD68 (+) inflammatory cells.

Restenosis after PTA can lead to a decrease in the lumen vessel area and VNH/VS. We assessed for this possibility by assessing vascular remodeling using histomorphometric analysis in 1,25 NP treated vessels compared to control controls. There was a significant increase in the average lumen vessel area with a decrease in N/M ratio and cell density in the neointima. These observations are consistent with our previous study in murine AVFs in which we administered 1,25 NP to adventitia of the outflow vein^7^. Because WSS and blood flow velocity have been hypothesized as factors leading to the development of VNH associated with AVF^15^, the changes in PV and WSS after PTA were determined. A previous study from our lab showed differences in WSS after PTA compared to control vessels and in this model, increased PV and WSS are seen in vein segments with positive vascular remodeling^16^. In the present study, we extended these results by performing Doppler US to assess the hemodynamic function of the AVF. We observed a significant increase in the average PV and WSS in 1,25 NP treated vessels compared to controls. The present model has increased WSS in the outflow vein in part due to the angle of the anastomosis, which is approximately 90 degrees and this angle has been shown to cause increased shear stress when compared to an angle of 45 degrees^17^.

Angioplasty can lead to VS formation due to an increase in α-SMA (+) cells accompanied with inflammatory cells and fibrosis. We assessed for changes in SMCs by staining for α-SMA. There was an increase in α-SMA staining in 1,25 NP treated vessels compared to controls. There are different phenotypes of SMCs. We assessed for the presence of contractile SMC phenotype by staining for MHY11 and synthetic SMCs by staining for vimentin, MMP-2, and MMP-9 staining^18^. Synthetic smooth muscles cells are hypothesized to cause restenosis after vascular injury^18^. Several cytokines have been shown to be involved including transforming growth factor-β^19^. We observed an increase in MHY11 (+) cells, which colocalized with α-SMA in 1,25 NP treated vessels. We next assessed for the presence of synthetic SMCs, and observed a significant reduction in in vimentin, MMP-2, and MMP-9 staining in 1,25 NP treated vessels compared to controls. The mechanism of how 1,25 NP modulates the phenotype of SMC by increasing the contractile SMCs while reducing synthetic SMCs is not well understood.

The vessel wall remodels to PTA by changes in fibrosis and deposition of extracellular matrix and thus reducing fibrosis and collagen formation after PTA and therefore this is a potential therapeutic strategy for improving outcomes. We assessed changes in fibrosis and collagen deposition by performing picrosirius red, Masson’s trichrome, and collagen staining after 1,25 NP treatment compared to controls. We observed a reduction in Picrosirius red (staining of collagen 1 and 3), Masson’s trichrome, and collagen 4 staining. These results imply that less constrictive remodeling is occurring after 1,25 NP treatment and this is allowing for improved positive vascular remodeling.

Because fibroblast to smooth muscle differentiation can cause to VS formation in murine AVF, we assessed the presence of fibroblasts using FSP-1 staining^16^. We observed a reduction in FSP-1 staining in 1,25 NP treated vessels compared to controls. This is consistent with previous study in murine AVFs using 1,25 NP^7^. Several studies have demonstrated that reduced FSP-1 staining is associated with improved vascular remodeling in experimental AVFs^20^. Moreover, there was no difference in FAP-1 staining indicating 1,25 NP treatment had no effect on fibroblast activation.

It is well known that angioplasty can lead to vascular injury, which causes inflammatory cells including macrophages and monocytes to accumulate in the vessel wall^12,21^. We assessed for the presence of inflammatory cells by performing staining for CD68 in PTA treated vessels compared to contralateral control veins. There was an increase in CD68 (+) cells in PTA treated vessels. Studies have demonstrated that reducing inflammation after vascular injury caused by PTA or stent placement is associated with improved patency^22,23^. We next assessed the presence of inflammatory cells by performing staining for CD68 (+), F4/80 (+), CD45 (+) cells. We observed that there was a significant reduction in CD68 (+), F4/80 (+), CD45 (+), and iNOS (+) cells in 1,25 NP treated vessels compared to controls. These findings are consistent with prior studies that showed that reducing CD68 (+) cells after PTA was associated with improved vascular remodeling and lumen vessel area^24,25^.

Several studies have shown that *Ier3* gene expression is involved with apoptosis^26^. We assessed for changes in apoptosis by performing TUNEL and cleaved caspase 3 staining after 1,25 NP treatment. We observed a significant increase in cells staining positive for TUNEL and cleaved caspase 3 staining. We further performed double staining to identify which cells were affected. Meanwhile there was increased TUNEL staining in both FSP-1 and CD68 (+) cells. Since we observed changes in apoptosis, we assessed proliferation by performing Ki-67 staining and observed it was significantly reduced in 1,25 NP treated vessels compared to vehicle controls. Next, we determined the phenotype of the cells undergoing proliferation and observed that proliferation was decreased in CD68, FSP-1, and SMCs in 1,25 NP treated vessels. We have previously reported that 1,25 NP decreases proliferation and increases cell death as assessed by TUNEL staining^7^. Collectively, these data imply that reduced proliferation with increase in apoptosis results in a decrease in cell density with a reduction in FSP-1 and CD68.

The potential changes in kidney and liver function after 1,25 NP delivery was evaluated by performing liver function test assessment measuring AST, ALT, total bilirubin and BUN and creatinine levels. Systemic administration of 1α,25(OH)_2_D_3_ can cause hypercalcemia^27^. In the present study, there was no difference between all these serologic measures at day 3 and 21 after delivery in the 1,25 NP and vehicle control groups.

Adventitial delivery of 1,25 NP has several advantages. It allows for the delivery of drug to the vessel wall with increased concentration and without the wash out of the drug as would be seen with endothelial delivery using drug coated balloons or stents^28^. Adventitial delivery is superior to systemic delivery as it reduces non-target delivery of the drug during the first pass effect by the liver^28^. We have utilized this approach for delivering cells, lentiviral agents, and drugs to the vessel wall for reducing vascular injury and VS formation^7,13,16,29^.

Finally, an unbiased whole transcriptomic analysis using RNA sequencing with Panther analysis was performed to identify mechanisms of 1,25 NP treatment. There were 311 genes that were upregulated and 874 genes that were downregulated after 1,25 NP delivery compared to vehicle controls. Panther analysis demonstrated that inflammatory pathways were involved in the upregulated genes and apoptotic pathways were involved in the down-regulated genes.

There are several limitations of the present study. First, we used a single dose; and thus, the effects of higher dose or multiple doses should be evaluated in the future. Second, these results need to be corroborated by using a larger animal model, as the murine model may not be the same as the clinical scenario. Changes in WSS and PV can occur as result of blood flow and mechanical factors. Drug treatment using 1,25 NP likely did not have a direct effect on WSS and PV changes. Rather, the changes in WSS and PV reflect the effects of 1,25 NP on reducing VNH.

In conclusion, we demonstrate that adventitial delivery of 1,25 NP to the outflow vein after PTA in a murine model results in a decrease in IER3 gene and protein expression. This was localized to CD68 (+) and FSP-1 (+) cells which have decreased proliferation and increased apoptosis. This leads to a reduction in pro-inflammatory macrophages, venous neointimal hyperplasia, accompanied by a decrease in cell proliferation and venous fibrosis with positive vascular remodeling. The clinical importance of this study is that this that it provides rationale for local drug delivery using 1,25 NP for improving interventional outcomes in hemodialysis patients after PTA.

## Materials and methods

### Patient population and endovascular biopsy

Mayo Clinic Institutional Review Board (IRB18-009466) approval was obtained prior to conducting the present study. All experiments were performed in accordance with relevant guidelines and regulations. Informed written consent was obtained from all patients with dysfunctional AVFs requiring an interventional procedure prior to performing endovascular biopsy of the stenosis and non-stenotic veins. Only patients > 18 years old were enrolled. A total of 5 patients (1 Female) underwent an angiogram with PTA of an AVF stenosis. The patient demographics and location of stenosis are presented (Supplemental Table 1). An angiogram was performed to identify a stenosis (Fig. 1A). Once the stenosis was identified, a 5F glide catheter (Boston Scientific, Natick, MA) was advanced across the stenosis carefully using a 0.035″ glide wire (Terumo Medical Corporation, Tokyo, Japan). The glide wire was removed and a 0.035″ Rosen wire (Cook Inc., Bloomington, IN) was advanced and used to gently cross the stenosis multiple times (Fig. 1B). The tip of the wire was withdrawn into the Glide catheter and removed as a unit. The tip of the wire was cut and placed in an RNA free plastic tube with 1 ml of lactated Ringer’s solution and placed on ice and saved for cell isolation and qRT-PCR analysis (see later). Figure 1C shows an angiogram prior to biopsy and Fig. 1D is after biopsy. A portion of non-stenotic vein from the fistula was sampled in an identical fashion to serve as control.

### Endothelial cell sorting

The wire fragment was removed and centrifuged at 1000 RPM for 10 min at 4 °C. The cell pellet was washed 3 times with 1 ml of PBS with 1% BSA. The pellet was resuspended in 100 µl of PBS with 1% BSA. CD31 (+) endothelial cells were sorted using magnetic beads. We added 10-ul of CD31 antibody (cat# 555444, BD Biosciences) to the cell suspension and it was incubated for 15 min at 4 °C. Next, anti-mouse IgG coupled magnetic beads (10ul, Cat# S1431S, New England Bio labs) were added to the suspension and CD31 (+) cells were sorted out. cDNA was synthesized directly using SuperScript III Cells Direct cDNA Synthesis Kit (cat# 18080200, Thermo Fischer).

### Mice study

Approval was obtained from the Mayo Clinic Institutional Animal Care and Use Committee prior to performing any experiments. All experiments were performed in accordance with relevant guidelines and regulations. All the authors complied with the ARRIVE guidelines experiments.

Eighteen C57BL/J6 male mice (Jackson Laboratories, Bar Harbor, ME) aged 6–8 weeks were housed at 12/12 h light/dark cycles, 22 °C, and 41% relative humidity with access to food and water ad libitum. Mice were randomly assigned to vehicle or 1,25 NP group (Supplementary Fig. 1). Mice were anesthetized using a combination of ketamine (100 mg/kg) and xylazine (10 mg/kg) administered by intraperitoneal injection prior to all procedures. Before surgery, one dose of buprenorphine-SR (0.05–0.1 mg/kg body sc) was administered for pain relief.

### Encapsulation of 1α,25(OH)_2_D_3_ in nanoparticles composed of PLGA and loaded in a Pluronic F127 Hydrogel

1α,25(OH)_2_D_3_ was encapsulated into nanoparticles composed of PLGA using the interfacial method and loaded into Pluronic F127 hydrogel as described previously by our laboratory^7^. Briefly, 100 mg of PLGA (Sigma-Aldrich, St. Louis, MO) and 0.1 mg of 1α,25(OH)_2_D_3_ (Tocris Bioscience, Bristol, UK) was dissolved in 10-mL of acetone. Then, the solution was added drop wise to 500 mL of deionized water at constant stirring. After particle formation, the organic solvent was evaporated and the PLGA nanoparticles were dried by lyophilization. The dried PLGA nanoparticles were dispersed in an aqueous solution to make a 200-µM solution. For vehicle controls, an equivalent amount of PLGA particles without 1α,25(OH)_2_D_3_ was used. A 40% aqueous solution of Pluronic F-127 (Sigma-Aldrich, St. Louis, MO) was prepared under sterile conditions at 4 °C. An equal portion of PLGA particles in water and equal portion of 40% Pluronic F-127 gel was mixed to obtain a final concentration of 100 µM, 1α,25(OH)_2_D_3_-PLGA nanoparticles in 20% hydrogel. We have previously showed that by day 3 and 28 the cumulative releases of 1α,25(OH)_2_D_3_ in our 1,25 NP drug was 40% and 80%, respectively^7^.

### Surgical creation of murine model

Chronic kidney disease was created by surgical ligation of the arterial blood supply to the upper pole of the left kidney accompanied by removal of the right kidney. We have used and described this model previously^4,11,16,30^ (Supplementary Figs. 1A,B). Four weeks later, an AVF was created by connecting the end of the right external jugular vein to the side of the left common carotid artery using an 11-0 nylon suture (Aros Surgical Instruments Corporation, Newport Beach, CA). Two weeks later, the diameter of the jugular vein was measured intraoperatively and PTA was performed using a 1.25 mm by 6-mm long balloon inflated to 14-atmospheres for 30-s (Medtronic Sprinter Legend, Minneapolis, MN)^4^. Next, 10-μL of either 1,25 NP in hydrogel or control vehicle (PLGA without 1,25 NP in hydrogel) was layered circumferentially onto the adventitia of the outflow vein for a length of 6-mm.

### Blood urea nitrogen (BUN), creatinine (Cr), aspartate aminotransferase (AST), alanine aminotransferase (ALT), total bilirubin, and calcium assay

Blood was removed to determine the kidney function, liver function tests, and calcium concentration at sacrifice at day 3 and 21 after PTA as described elsewhere^31^. A QuantiChrom Urea Assay Kit (BioAssay Systems, Hayward, CA) and Mouse Creatinine Assay Kit (Crystal Chem, Elk Grove Village, IL) were used to determine the serum BUN and Cr, respectively. The Preventive Care Profile Plus rotor (Abaxis, Union City, CA) was used in a Vetscan VS2 machine (Abaxis, Union City, CA) to determine the AST, ALT, total bilirubin, and calcium levels^31^.

### Doppler ultrasound (US) examination

As described previously, a high-frequency 20-MHz transducer probe (Doppler Flow Velocity System, INDUS Instruments, Houston, TX) was used to evaluate the AVF patency and blood flow velocity^4,11,16,30^. Eight consecutive cycles were recorded. Murine peak velocity data were analyzed using a Doppler signal processing workstation (Doppler Flow Velocity System version 1.627, INDUS Instruments) in the murine peripheral blood flow mode. The average WSS was calculated by the equation *WSS* = 4η*V*/*r,* where η is blood viscosity, V is velocity (m/s), and r is the radius obtained from intraoperative measurements of the outflow vein (m). The viscosity of blood was assumed to be constant at is the viscosity (0.00345 4 N s m^−2^)^32^.

### Murine tissue collection and processing

At 3 days after PTA, mice were sacrificed for gene and RNA sequencing expression analysis, and the outflow vein samples were stored in RNA later solution (Qiagen, Hilden, Germany). Twenty-one days after PTA, we dissected the outflow veins and fixed the tissue in 10% formalin reagent (Fisher Scientific, Pittsburgh, PA) using a non-pressurized method of fixation. The vessels were analyzed for histomorphometric and immunobiological analyses. Each vessel was embedded in paraffin lengthwise as described previously. Typically, an average of 60–80 consecutive 4-μm sections were obtained per outflow vein per animal for analysis.

### RNA isolation and cDNA synthesis

RNA was isolated using miRNeasy kit (Qiagen) from all mice tissue samples and cDNA was synthesized using iScript kit (BioRad, Hercules, CA) according to the manufacturer’s instructions.

### Quantitative real time polymerase chain reaction (qRT-PCR)

The endothelial cells were isolated from each patient’s sample and analyzed for gene expression of *Ier3* using quantitative real time polymerase chain reaction (qRT-PCR) as described previously^20^. At day 3, 1,25 NP or vehicle treated murine outflow veins were analyzed for gene expression of *Ier3* and *Ccl2*. The primer design information is shown in Supplementary Table 3. All qRT-PCRs were performed using an iTaq universal SYBR Green Master Mix (BioRad) in a C1000 thermal cycler equipped with a CFX96 Real Time System (BioRad) and cq values were measured using BioRad CFX Manager software (BioRad). The ∆cq values of human were normalized to 18S. For mouse vein samples, ∆cq values were normalized to TBP1 and the fold change in gene expression was calculated using the 2^−(ΔΔCT)^ method.

### RNA sequencing and bioinformatics analysis

Whole transcriptomic mRNA-Seq was performed at the Mayo Clinic Medical Genome Facility^30^. RNA libraries were prepared using the TruSeq RNA sample kit V2 (Illumina, San Diego, CA) following the manufacturer’s protocol. mRNA libraries were loaded into flow cells (TruSeq v3 paired-end flow cells; Illumina) at concentrations designed to generate 100 million total reads/sample following Illumina’s standard protocol using the Illumina cBot and TruSeq Paired end cluster kit version 3. The flow cells were then sequenced on HiSeq 2000/2500 using the TruSeq SBS sequencing kit version 3 (Illumina) and the data was collected using HiSeq data collection version 2.0.12.0 software. Base-calling was performed using Illumina’s RTA version 1.17.21.3. Data analysis was further performed using the MAPRSeq v.1.2.1 system, the Bioinformatics Core standard tool, TopHat 2.0.6 and Feature Counts software^11^. Gene expression was standardized to 1 million reads and normalized for gene length (reads per kilobase pair per million mapped reads, RPKM). The mRNAs with a fold change of > 1.5 and < 0.75 and an average RPKM > 0.01 were considered up regulated and down regulated, respectively, in 1,25 NP treated or vehicle control tissue. A student t-test was performed and the difference in the fold change was considered statistically significant if *p* < 0.05. The mRNA list was then uploaded to Gene-E (https://software.broadinstitute.org/GENE-E) to create a heat map. PANTHER (http://pantherdb.org/) was performed for pathway analysis.

### Immunohistochemistry and immunofluorescence staining

Staining was performed on paraffin-embedded outflow vein sections from 1,25 NP and vehicle treated groups following the EnVision (Dako, CA) method with a heat-induced antigen-retrieval step^16^. The primary antibodies used are listed in Supplementary Table 4. Peroxidase activity was visualized using 3,3′-diaminobenzidine as chromogen. Immunoglobulin G (IgG) stained slides served as negative controls. Prolong Gold anti-fade reagent with DAPI (Invitrogen, Eugene, OR) was used for nuclear staining and mounting for immunofluorescence staining.

### TUNEL, Masson’s trichrome, Picrosirius red and Alizarin Red S staining

TUNEL staining was performed on paraffin-embedded sections to evaluate apoptosis according to the manufacturer’s instructions (TACS 2 TdT DAB in situ Apoptosis Detection Kit, Thermo Scientific, Waltham, MD). Slides without TdT enzyme were used as negative controls^16^. Picrosirius red (Sigma-Aldrich, St. Louis, MO) staining was performed to evaluate for vascular fibrosis and collagen 1 and 3 by analyzing the slides under polarized light Alizarin Red S (Spectrum, Gardena, CA) staining was performed to evaluate for vascular calcification. Masson’s trichrome was performed as described previously on unstained paraffin embedded sections^16^.

### Morphometric and image analysis

We analyzed 3–5 outflow veins per animal per group for histomorphometric analysis. Sections were stained for Hematoxylin and Eosin (H and E) to assess venous remodeling. Images were digitized to capture a minimum of 1936 × 1460 pixels covering one entire cross-section utilizing an M2 Microscope (Carl Zeiss) with an Axiocam 503 color camera (Carl Zeiss). These slides were analyzed using ZEN 2 blue edition version 2.0 (Carl Zeiss) as described elsewhere^4,11,16,30^. We measured the lumen vessel area, neointima, media, and adventitia areas, along with cell density in each of the layers.

### Semiquantification of immunobiological stains and immunofluorescent stains

Sections that were stained with immunobiological stains and immunofluorescent stains were digitized to capture a minimum of 1936 × 1460 pixels covering one entire cross-section utilizing an M2 Microscope (Carl Zeiss) with an Axiocam 503 color camera (Carl Zeiss). These slides were analyzed using ZEN 2 blue edition version 2.0 (Carl Zeiss) as described elsewhere^4,11,16,30^. Staining index was obtained by number of positive cells/ total number of cells × 100.

### Statistical analysis

Results are presented as the mean ± SEM. Statistical differences were tested by using either a one-way or two-way analysis of variance (ANOVA) followed by an unpaired Student *t* test with post hoc Bonferroni's correction. The level of significance was set at *NS p* > 0.05, *p < 0.05, **p < 0.01, ***p < 0.001 or ^#^*p* < 0.0001. GraphPad Prism version 8 (GraphPad Software Inc., La Jolla, CA) was used for all statistical analysis.

## Supplementary Information

Supplementary Figures.

Supplementary Information.

## Data Availability

The RNA seq dataset generated during and analyzed during the current study will be made available on the GEO website at https://www.ncbi.nlm.nih.gov/geo once the manuscript has been published. The remaining datasets generated during and/or analyzed during the current study are not publicly available due to because of the multiple different immunohistologic measurements, gene expression, serum, and histomorphometric analyses but are available from the corresponding author on reasonable request.
