# Supplementary material for: Adventitial delivery of nanoparticles encapsulated with 1α, 25-dihydroxyvitamin D3 attenuates restenosis in a murine angioplasty model
Source: Sci Rep. 2021 Feb 26;11:4772. doi: 10.1038/s41598-021-84444-x (PMC7910622; doi:10.1038/s41598-021-84444-x)

# **Adventitial delivery of nanoparticles encapsulated with 1α, 25-dihydroxyvitamin $D_3$ attenuates restenosis in a murine angioplasty model**

Chuanqi Cai,[1,2] Sreenivasulu Kilari,[2] Chenglei Zhao, [2,3] Avishek K Singh,[2] Michael L Simeon,[2] Avanish Misra,[2] Yiqing Li,[1] Edwin Takahashi, [6] Rajiv Kumar,[4,5] and Sanjay Misra[2,4,6]

[1]Department of Vascular Surgery, Union Hospital, Tongji Medical College, Huazhong University of Science and Technology, Wuhan, 430022, China; [2]Vascular and Interventional Radiology Translational Laboratory, Department of Radiology, Mayo Clinic, Rochester, MN, USA; [3]Department of Vascular Surgery, The Second Xiangya Hospital, Central South University, Changsha, Hunan, China; [4]Department of Biochemistry and Molecular Biology, Mayo Clinic, Rochester, MN, USA; [5]Department of Internal Medicine, Division of Nephrology and Hypertension, Mayo Clinic, Rochester, MN, USA; [6]Department of Radiology, Vascular and Interventional Radiology, Mayo Clinic Rochester, MN, USA.

**Address correspondence to:**

Sanjay Misra, MD, FSIR, FAHA
Professor of Radiology
Department of Radiology
Mayo Clinic
200 First St SW
Rochester, MN 55905
Telephone: 507-293-3793
Fax: 507-255-7872
Email: misra.sanjay@mayo.edu

Supplementary Figure 1

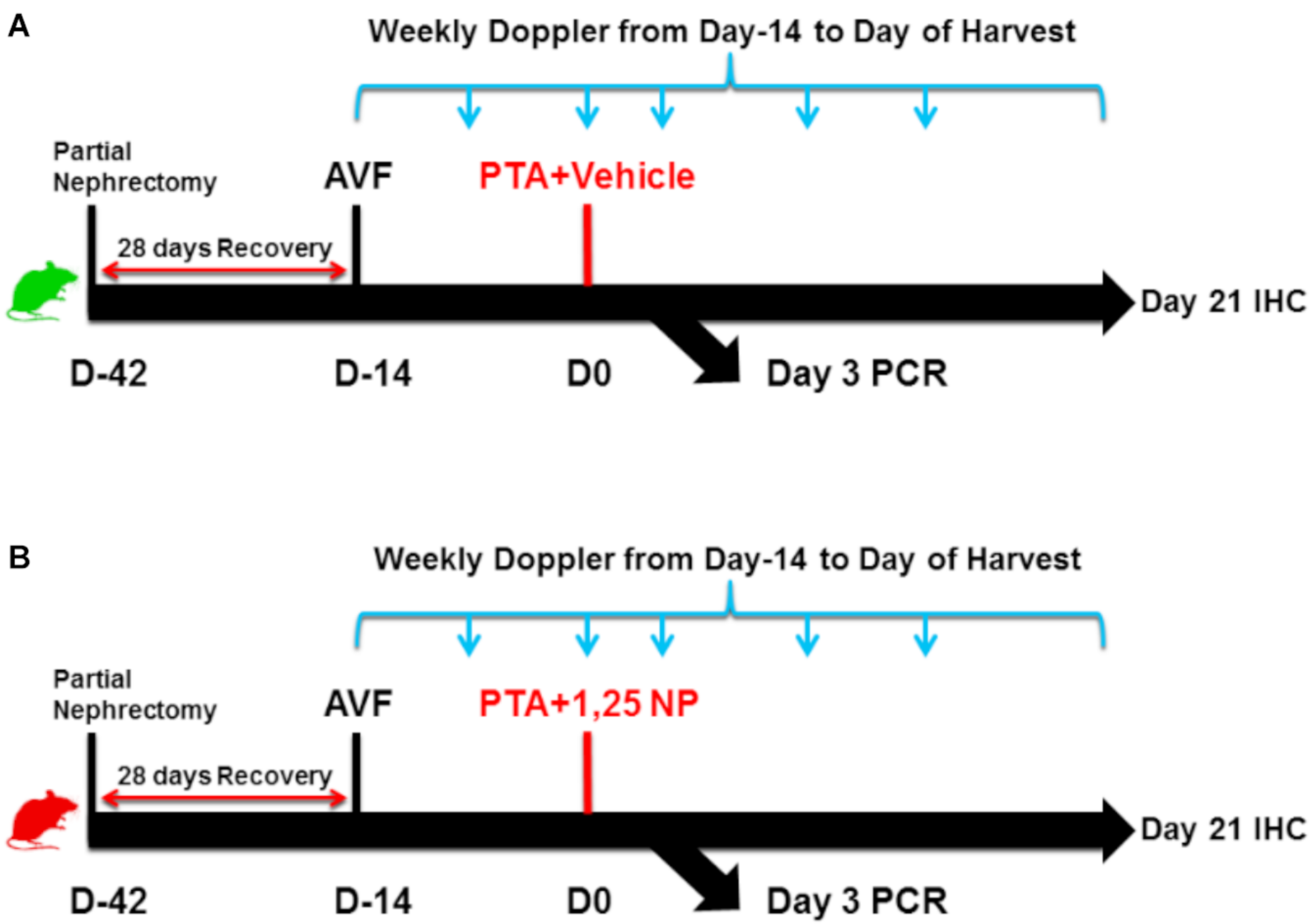

Supplementary Figure 2

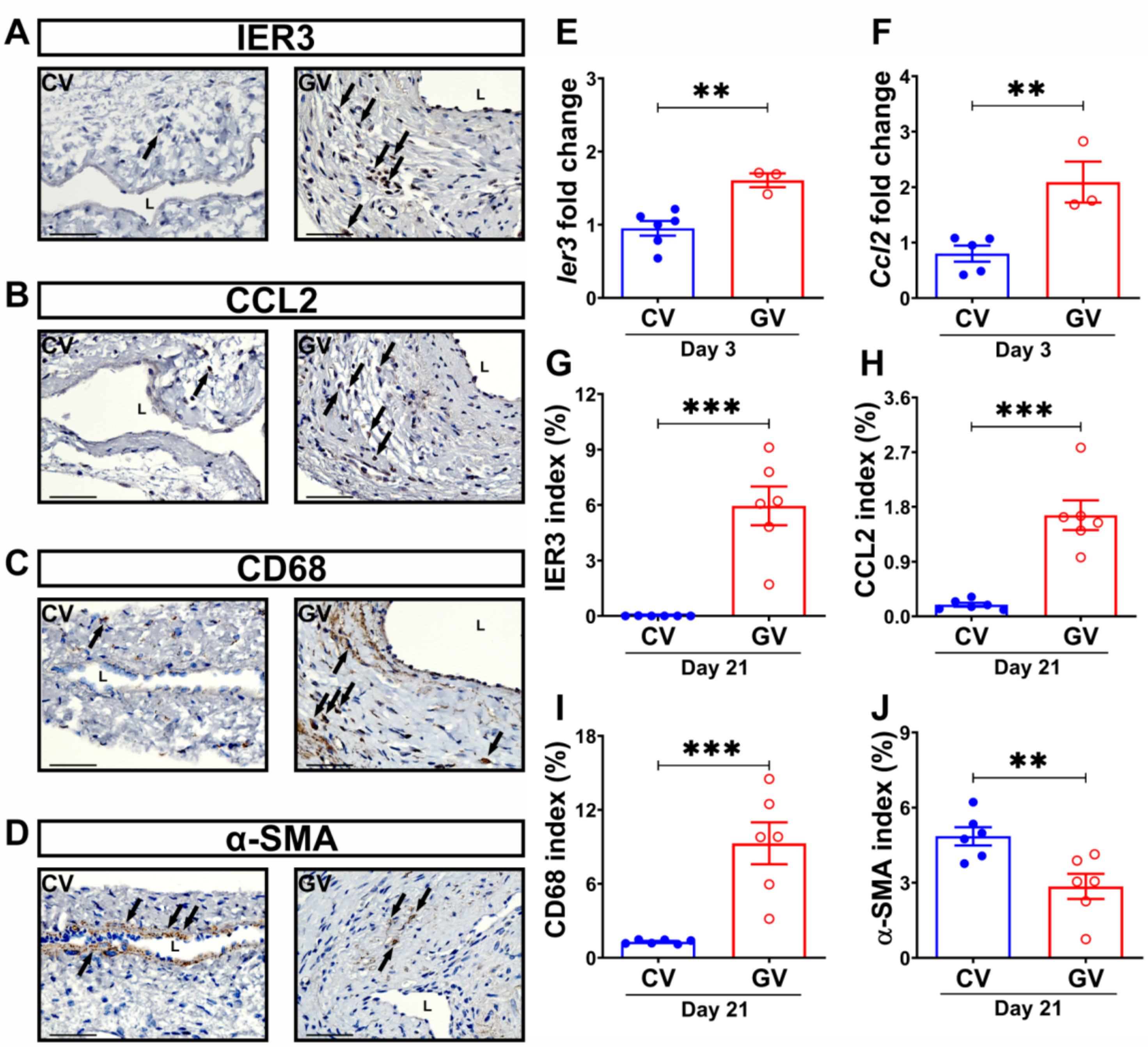

Supplementary Figure 3

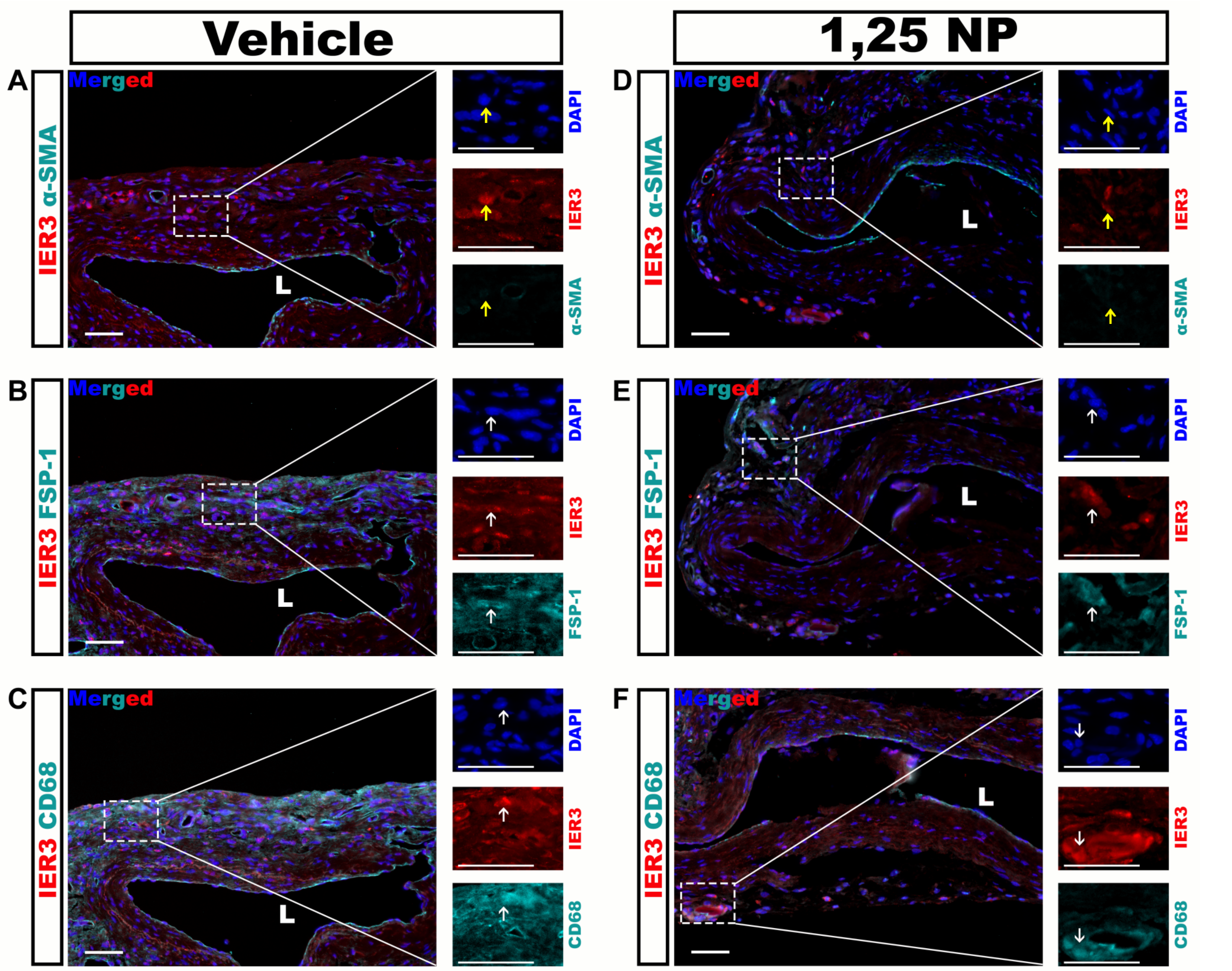

Supplementary Figure 4

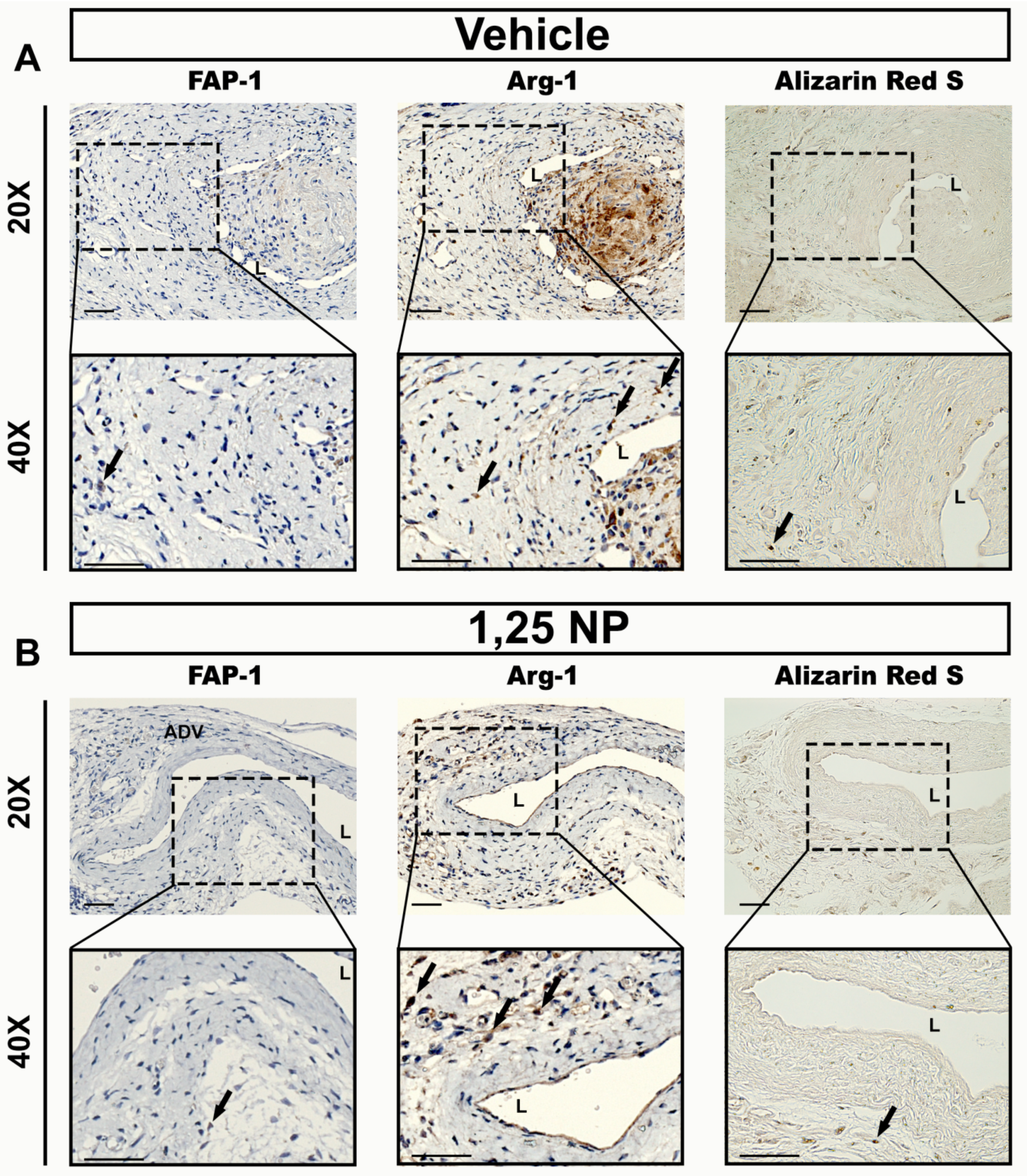

Supplementary Figure 5

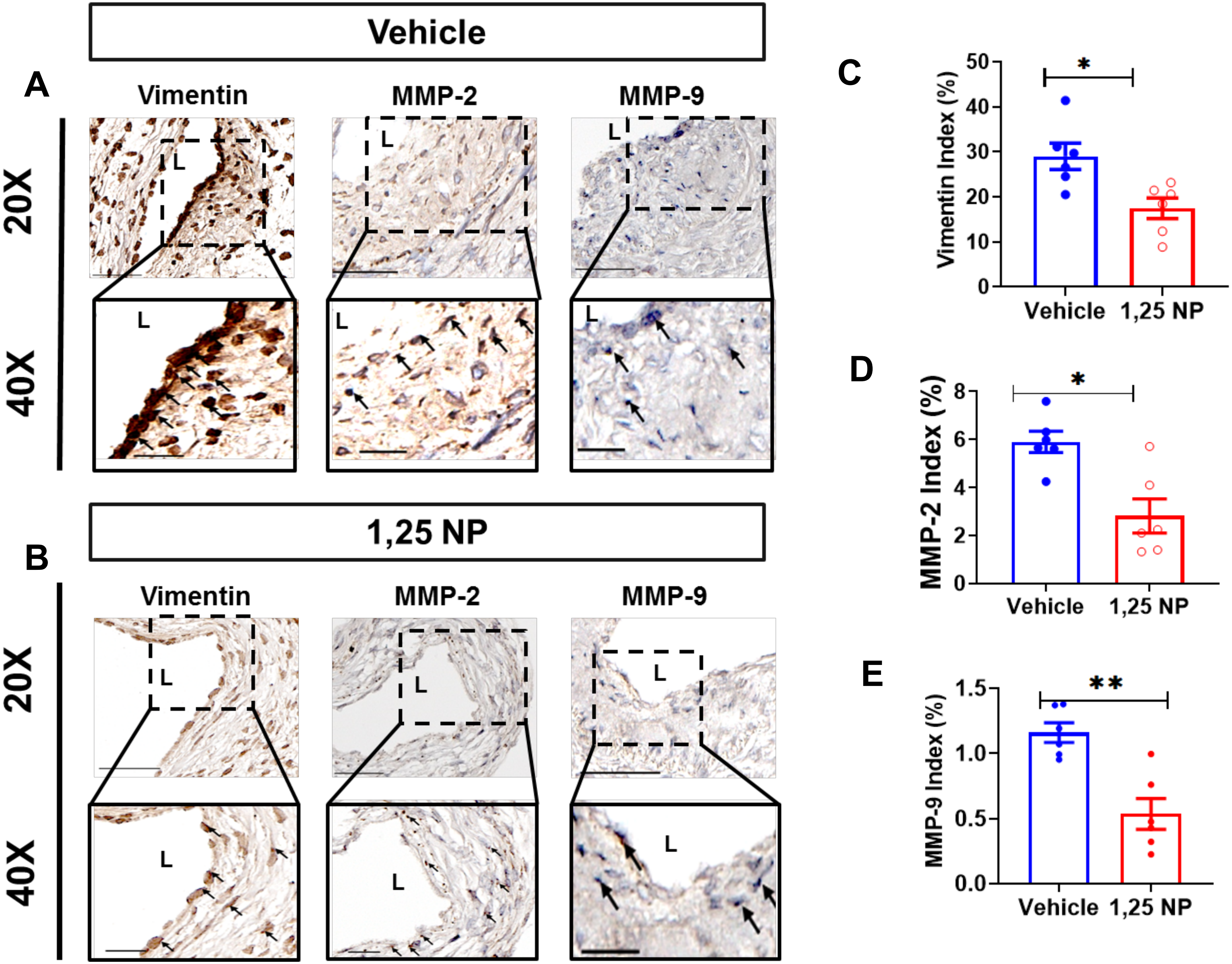

Supplementary Figure 6

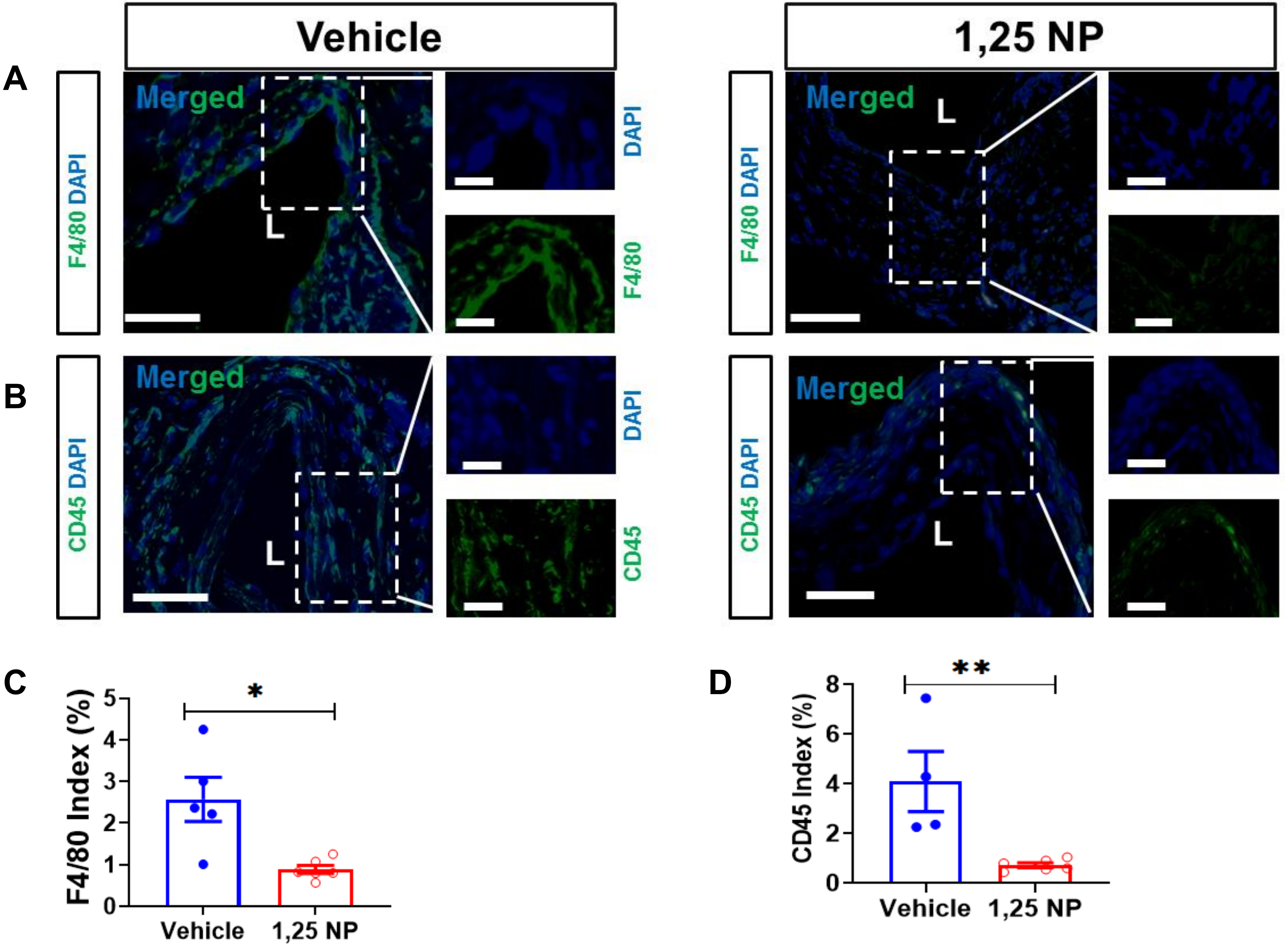

Supplementary Figure 7

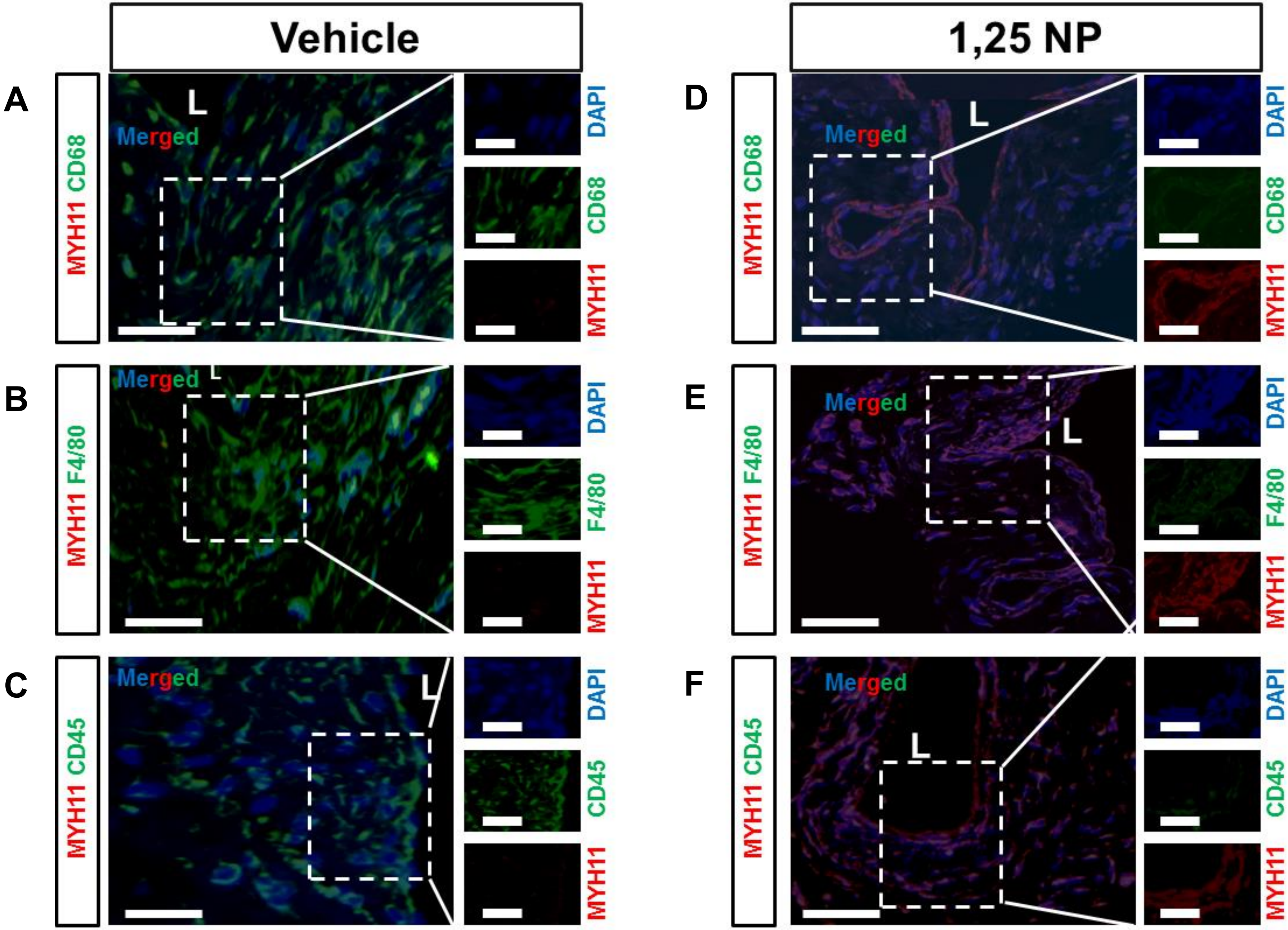

Supplementary Figure 8

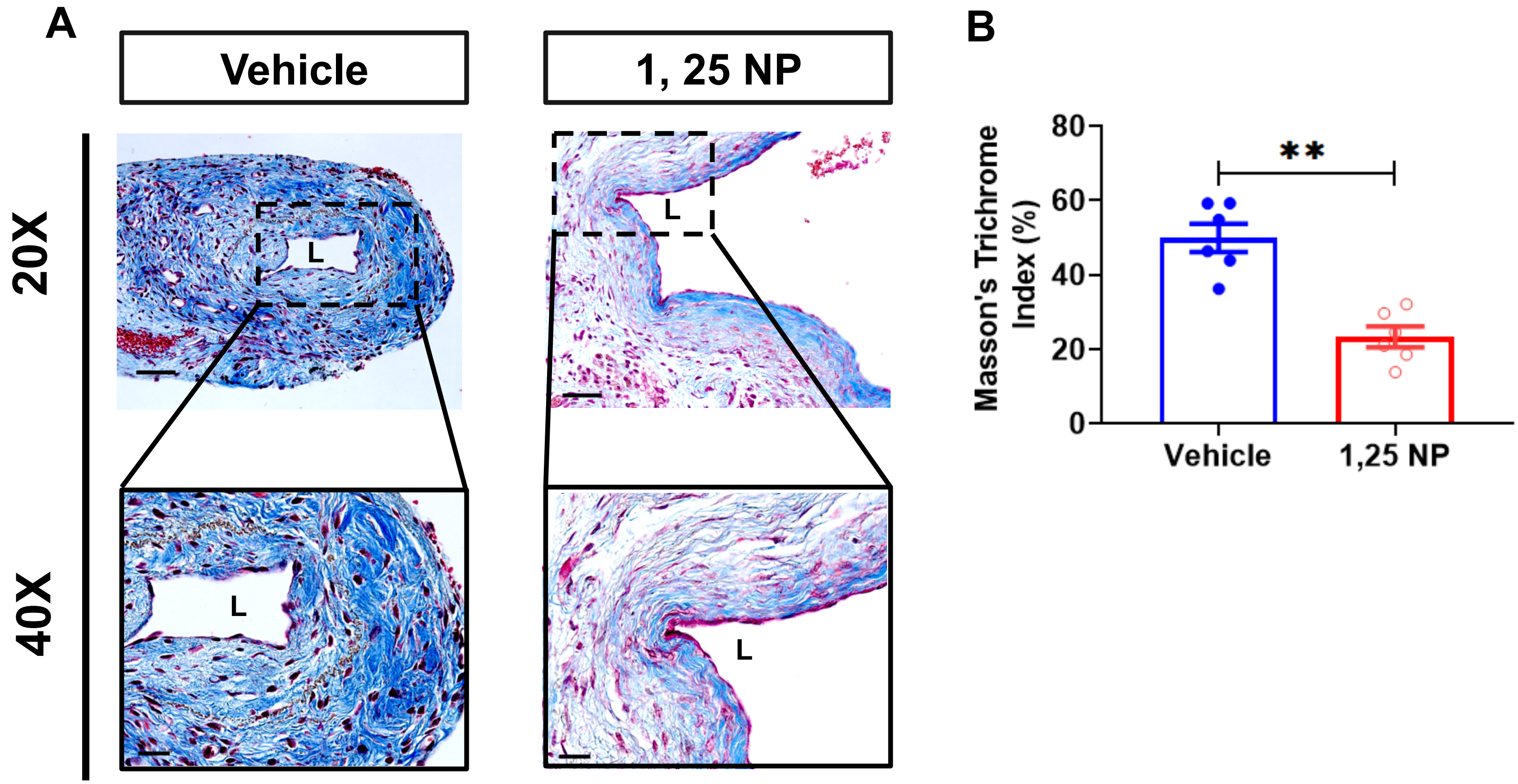

Supplementary Figure 9

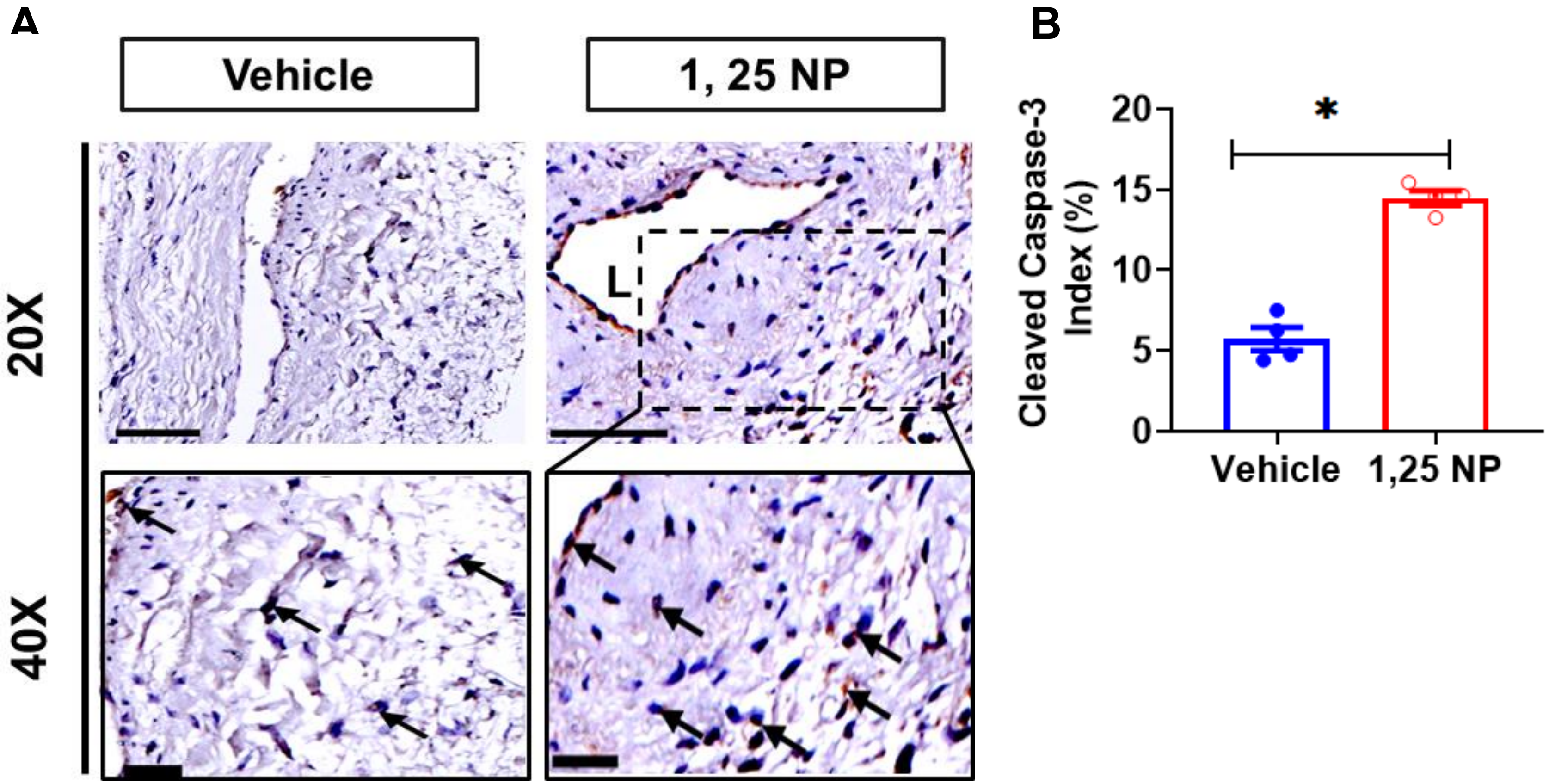

Supplementary Figure 10

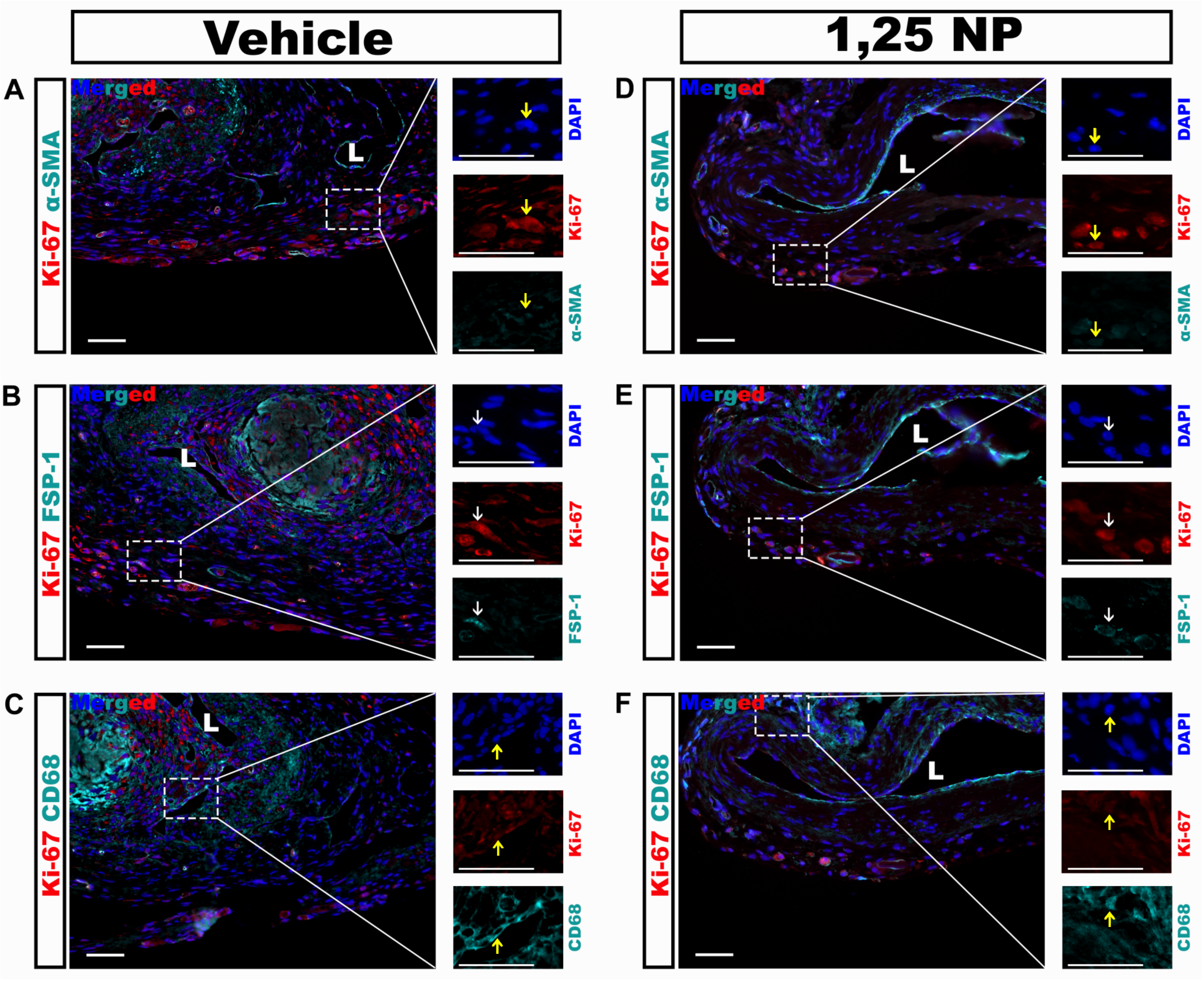

Supplementary Figure 11

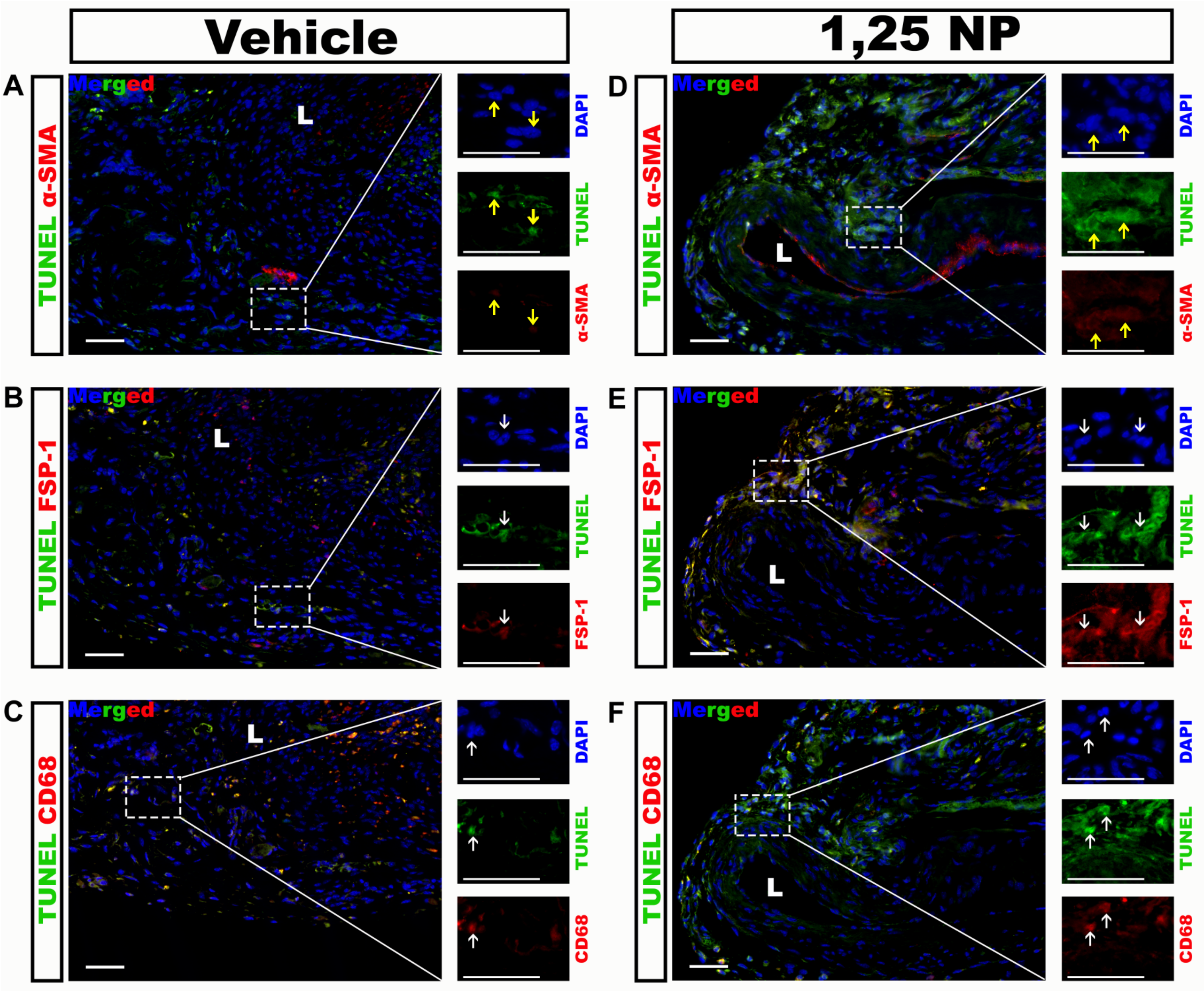

Supplement: Supplementary file 1 — Supplementary Figures. [file 41598_2021_84444_MOESM1_ESM.pdf]
