# Supplementary material for: Adventitial delivery of nanoparticles encapsulated with 1α, 25-dihydroxyvitamin D3 attenuates restenosis in a murine angioplasty model
Source: Sci Rep. 2021 Feb 26;11:4772. doi: 10.1038/s41598-021-84444-x (PMC7910622; doi:10.1038/s41598-021-84444-x)
Supplement: Supplementary file 2 — Supplementary Information. [file 41598_2021_84444_MOESM2_ESM.docx]

**Supplementary Figure 1. Outline of PTA plus perivascular delivery study.**

(A and B) Schematic of PTA +vehicle and PTA + 1,25 NP mice, Doppler detection was performed weekly.

**Supplementary Figure 2. Increased IER3 gene and protein expression in PTA treated vessels.**

(A-D) Representative IER3, CCL2, CD68, and α-SMA staining at day 21 in the PTA treated outflow graft veins (GVs) and contralateral jugular veins (CVs), respectively. (E-F) At day 3, there was a significant increase in the average gene expression of *Ier3* and *Ccl2* of PTA treated GVs compared to CVs, respectively. (G-I) Semiquantitative analysis demonstrated a significant increase in the average IER3, CCL2 and CD68 indexes in GVs compared to CVs. (J) There was a significant decrease in α-SMA staining after PTA treatment at day 21. Each data point in the scatter plot bar graph represents the mean ± SEM of 3-6 animals. Unpaired *t-*test was performed. Significant differences between groups are indicated **p*<0.05, ***p*<0.01, ****p*<0.001. Cells staining positive for IER3, CCL2, α-SMA and CD68 are brown staining. CV, contralateral jugular vein; GV, outflow graft vein. L, lumen; solid arrows, positive cells. Scale bar is 50μm.

**Supplementary Figure 3. IER3 co-staining with α-SMA, FSP-1 and CD68 cells.**

(A, B and C) Representative images of IER3 co-staining with α-SMA, FSP-1 and CD68 showed positive IER3 staining was co-localized to FSP-1 and CD68 (+) cells but not α-SMA (+) cells in the vehicle control group. (D, E and F) Representative images from 1,25 NP treated vessels demonstrates decreased expression of IER3 in FSP-1 (+) and CD68 (+) cells but not α-SMA (+) cells. Turquoise color indicates positive α-SMA, FSP-1 or CD68 staining. Red color indicates IEX-1 positive staining. Blue color indicates positive staining for nuclei. L, lumen; yellow solid arrows, non-co-staining cells; white solid arrows, positive co-stained cells. Scale bar is 50μm.

**Supplementary Figure 4. FAP-1, Arg-1 and Alizarin Red S staining for vehicle and 1,25 NP treated vessels.**

(A and B) Representative images of FAP-1, Arg-1 and Alizarin Red S staining in vehicle control and 1,25 NP treated vessels. There was no significant difference in the average FAP-1, Arg-1 and Alizarin Red S staining between two groups. Positive FAP-1 and Arg-1 staining are brown staining. Positive Alizarin Red S staining is dark pink staining. ADV, adventitial; L, lumen; black solid arrows indicate positive staining. Scale bar is 50μm.

**Supplementary Figure 5. Vimentin, MMP-2, and MMP-9 staining in vehicle and 1,25 NP treated vessels.**

(A and B) Representative images for vimentin, MMP-2, and MMP-9 staining are shown from vehicle controls and 1,25 NP treated vessels. Cells staining positive for vimentin, MMP-2 and MMP-9 are brown staining. (C, D and E) Semiquantitative analysis showed a significant decrease in the average vimentin (*p*<0.05), MMP-2 (*p*<0.05) and MMP-9 (*p*<0.01) staining in 1,25 NP treated vessels compared to vehicle controls. Each data point in the scatter plot bar graph represents the mean ± SEM of 5-6 AVF mice. Unpaired *t-*test was performed. Significant differences are indicated **p*<0.05; ***p*<0.01. L, lumen; Scale bar is 50μm.

**Supplementary Figure 6. F4/80 and CD45 Immunofluorescence staining for vehicle and 1,25 NP treated vessels.**

(A and B) Representative images of F4/80 and CD45 immunofluorescence staining are shown from vehicle controls and 1,25 NP treated vessels. Positive F4/80 and CD45 staining are green. Blue color indicates positive staining for nuclei. L, lumen; Scale bar is 50μm. (C and D) Semiquantitative analysis demonstrates a significant decrease in the average F4/80 (*p<*0.05) and CD45 staining (*p*<0.01) in 1,25 NP treated vessels compared to vehicle controls. Each data point in the scatter plot bar graph represents the mean ± SEM of 4-6 AVF mice. Unpaired *t-*test was performed. Significant differences are indicated **p*<0.05 and ***p*<0.01. Scale bar is 50μm

**Supplementary Figure 7. MYH11 co-staining with CD68, F4/80, and CD45 cells**

(A, B and C) Representative images of MYH11 co-staining with CD68, F4/80 and CD45 cells are shown from vehicle controls and 1,25 NP treated vessels. Red color indicates positive MYH11 staining. Green color indicates positive CD68, F4/80 or CD45 staining. Blue color indicates positive staining for nuclei. MYH11 (+) cells did not co-localize with CD68 (+) cells, F4/80 (+) cells and CD45 (+) cells. (D, E and F) Representative images of MYH11 co-staining with CD68, F4/80 and CD45 cells in the 1,25 NP group. MYH11 (+) cells did not co-localize with CD68 (+) cells, F4/80 (+) cells and CD45 (+) cells. Scale bar is 50μm.

**Supplementary Figure 8. Masson’ Trichrome staining in vehicle and 1,25 NP treated vessels.**

(A) Representative slides for Masson’s Trichrome staining are shown from vehicle controls and 1,25 NP treated vessels. Collagen (blue color) is positive for Masson’s trichrome staining. (B) Semiquantitative analysis of collagen using Masson’s trichrome staining shows a significant reduction in the 1,25 NP group compared with the vehicle group (*p*<0.01). Each data point in the scatter plot bar graph represents the mean ± SEM of 5-6 AVFs. Unpaired *t-*test was performed. Significant differences are indicated ***p*<0.01. L, lumen; Scale bar is 50μm.

**Supplementary Figure 9. Cleaved Caspase-3 staining in vehicle and 1,25 NP treated vessels.**

(A) Representative slides for cleaved caspase-3 staining are shown from vehicle controls and 1,25 NP treated vessels. Positive cleaved caspase-3 staining is brown. (B) Semiquantitative analysis staining of cleaved caspase-3 shows a significant increase in the 1,25 NP group compared with the vehicle group (*p*<0.05). Each data point in the scatter plot bar graph represents the mean ± SEM of 5-6 AVFs. Unpaired *t-*test was performed. Significant differences are indicated **p*<0.05. L, lumen; Scale bar is 50μm.

**Supplementary Figure 10. Ki-67 co-staining with α-SMA, FSP-1 and CD68.**

(A, B and C) Representative images of Ki-67 co-staining with α-SMA, FSP-1 and CD68 showed positive Ki-67 staining was co-localized to FSP-1 (+) cells but not α-SMA and CD68 staining in vehicle vessels. (D, E and F) Representative 1,25 NP treated vessels showed less co-staining of Ki-67 (+) /FSP-1 (+), few Ki-67 (+)/CD68 (+) and Ki-67 (+)/α-SMA (+). Turquoise color indicates positive α-SMA, FSP-1 or CD68 staining. Red color indicates Ki-67 (+) staining. Blue color indicates positive staining for nuclei. L, lumen; yellow solid arrows, negative co-stained cells; white solid arrows, positive co-stained cells. Scale bar is 50μm.

**Supplementary Figure 11. TUNEL co-staining with α-SMA, FSP-1 and CD68.**

(A, B and C) Representative images of TUNEL co-staining with α-SMA, FSP-1 and CD68 showed positive TUNEL staining was co-localized with FSP-1 (+) cells and CD68 (+) cells but not α-SMA in vehicle vessels. (D, E and F) Representative 1,25 NP treated vessels showed more co-staining of TUNEL (+)/FSP-1 (+) and TUNEL (+)/CD68 (+) but not for TUNEL (+)/α-SMA (+). Green color indicates positive TUNEL staining. Red color indicates positive α-SMA, FSP-1 or CD68 staining. Blue color indicates positive staining for nuclei. L, lumen; yellow solid arrows, negative co-stained cells; white solid arrows, positive co-stained cells. Scale bar is 50μm.

**Supplementary Table 1. Baseline characteristics of the study participants**

| **Clinical factors (n=5)** | |  | |  |
| --- | --- | --- | --- | --- |
| Age, yr | | 66 ± 7 | |  |
| Sex, male | | 4 (80%) | |  |
| Hypertension | | 5 (100%) | |  |
| Diabetes mellitus | | 2 (40%) | |  |
| Dyslipidemia | | 5 (100%) | |  |
| Active smoker | | NA | |  |
| Coronary artery disease | | 4 (80%) | |  |
| Antiplatelet | | 5 (100%) | |  |
| ACEI / ARB | | 0 (0%) | |  |
| Statin | | 5 (100%) | |  |
| **Access and lesion factors** | |  | |  |
| Brachiocephalic fistula | | 4 (80%) | |  |
| Radiocephalic fistula | | 1 (20%) | |  |
| Right arm access | | 2 (40%) | |  |
| **Source of biopsy** | |  | |  |
| AV anastomosis | | 2 (40%) | |  |
| Cephalic arch | | 3 (60%) | |  |
|  |  |  |  |  |
|  |  |  |  |  |

Age is expressed as mean ± SD. The other parameters are expressed as n (percentage). NA, not applicable; ACEI/ARB, angiotensin-converting enzyme inhibitor / angiotensin II receptor blocker.

**Supplementary Table 2: Serum BUN, creatinine, AST, ALT, total bilirubin, and calcium over time**

 A. Serum BUN (mmol/L):

|  | 3 | 21 |
| --- | --- | --- |
| Vehicle | 22.08 ± 4.01 | 14.4 ± 1.49 |
| 1,25 NP | 0.28 ± 0.04 | 15.18 ± 2.21 |

B. Serum creatinine (µmol/L):

|  | 3 | 21 |
| --- | --- | --- |
| Vehicle | 38.83 ± 9.31 | 43.4 ± 15.63 |
| 1,25 NP | 24.5 ± 2.38 | 30 ± 2.59 |

C. ALT (U/L):

|  | 3 | 21 |
| --- | --- | --- |
| Vehicle | 23 ± 1.98 | 43.4 ± 15.63 |
| 1,25 NP | 24.5 ± 2.38 | 49.6 ± 17.68 |

D. AST (U/L):

|  | 3 | 21 |
| --- | --- | --- |
| Vehicle | 44.67 ± 3.48 | 59.6 ± 12.17 |
| 1,25 NP | 46.5 ± 2.95 | 55.75 ± 17.76 |

E. Total bilirubin (mg/dL):

|  | 3 | 21 |
| --- | --- | --- |
| Vehicle | 0.27 ± 0.02 | 0.28 ± 0.02 |
| 1,25 NP | 0.27 ± 0.02 | 0.28 ± 0.02 |

 F. Calcium (mg/dL):

|  | 3 | 21 |
| --- | --- | --- |
| Vehicle | 10.95 ± 0.29 | 10.1 ± 0.32 |
| 1,25 NP | 12.63 ± 0.80 | 10.48 ± 0.43 |

**Supplementary Table 3. qRT-PCR primer design**

| **Gene** | **Forward** | **Reverse** |
| --- | --- | --- |
| *Ier3* | GGGCACCTAGAGAATAACCAATC | ACACACCTTCTTACACCATTCC |
| *Ccl2* | GTCCCTGTCATGCTTCTGG | GCTCTCCAGCCTACTCATTG |
| TBP1 | AAGGGAGAATCATGGACCAG | CCGTAAGGCATCATTGGACT |
| *18S*  *Ier3* | **Human**  GTTCCGACCATAAACGATGCC  TTCTCTACCCTCGAGTGGTC | TGGTGGTGCCCTTCCGTCAAT  CACACCCTCTTCAGCCATC |

**Supplementary Table 4. Antibodies used in present study**

| **Antibodies** | **Host** | **Catalog number** | **Provider** | **Dilution** |
| --- | --- | --- | --- | --- |
| IgG | Rabbit | sc-2027 | Santa Cruz |  |
| CD68 | Mouse | ab955 | Abcam | 1:2000 |
| CD68 | Rabbit | ab125212 | Abcam | 1:2000 |
| α-SMA | Mouse | ab7817 | Abcam | 1:400 |
| α-SMA | Rabbit | ab5694 | Abcam | 1:1000 |
| iNOS | Rabbit | NB300-605 | Novus Biologicals | 1:2000 |
| Arg-1 | Rabbit | BNP1-32731 | Novus Biologicals | 1:1500 |
| FAP-1 | Rabbit | ab207178 | Abcam | 1:500 |
| IER3 | Rabbit | ab170453 | Abcam | 1:150 |
| CCL2 | Rabbit | ab25124 | Abcam | 1:100 |
| MYH11 | Rabbit | ab53219 | Abcam | 1:1000 |
| FSP-1 | Mouse | 188-11191 | RayBiotech | 1:500 |
| FSP-1 | Rabbit | 07-2274 | EMD Millipore | 1:1000 |
| Ki-67 | Rabbit | ab9260 | EMD Millipore | 1:350 |
| Collagen IV | Rabbit | 600-401-106 | Rockland | 1:2000 |
| Cleaved Caspase-3 | Rabbit | 9661 (Asp 175) | Cell Signaling | 1:1000 |
| F4/80 | Rat | 123103 | Biolegend | 1:100 |
| CD45 | Rat | 103129 | Biolegend | 1:100 |
| Vimentin | Mouse | ab8978 | Abcam | 1:250 |
| MMP-2 | Rabbit | ab79781 | Abcam | 1:600 |
| MMP-9 | Rabbit | Ab8898 | Abcam | 1:1500 |
| Alexa Fluor 488 | Goat | A11006 | Invitrogen | 1:1000 |
| Alexa Fluor 488 | Donkey | 711-545-152 | Jackson ImmunoResearch | 1:1000 |
| Alexa Fluor 594 | Donkey | 715-585-151 | Jackson ImmunoResearch | 1:1000 |
| Alexa Fluor 647 | Donkey | 715-605-151 | Jackson ImmunoResearch | 1:1000 |
| Alexa Fluor 647 | Goat | A32728 | Invitrogen | 1:1000 |
